# L‐arginine ameliorates hypertension and cardiac mitochondrial abnormalities but not cardiac injury in male metabolic syndrome rats

**DOI:** 10.14814/phy2.70183

**Published:** 2025-02-20

**Authors:** Kaito Tagami, Touko Okuzawa, Keisuke Yoshida, Rin Mishima, Natsuki Obara, Asuko Kunimatsu, Mayako Koide, Tamami Teranishi, Koji Itakura, Katsuhide Ikeda, Toyoaki Murohara, Kohzo Nagata

**Affiliations:** ^1^ Pathophysiology Sciences, Department of Integrated Health Sciences Nagoya University Graduate School of Medicine Nagoya Japan; ^2^ Department of Medical Technology Nagoya University School of Health Sciences Nagoya Japan; ^3^ Division for Medical Research Engineering Nagoya University Graduate School of Medicine Nagoya Japan; ^4^ Department of Cardiology Nagoya University Graduate School of Medicine Nagoya Japan

**Keywords:** cardiac injury, hypertension, L‐arginine, metabolic syndrome, mitochondria

## Abstract

L‐Arginine supplementation has beneficial effects on metabolic disorders in rodents. We here investigated the effects of exogenous L‐arginine on cardiac pathology and mitochondrial reactive oxygen species (ROS) production and dynamics in DahlS.Z‐*Lepr* ^*fa*^ */Lepr* ^*fa*^ (DS/obese) rats, a model of metabolic syndrome (MetS). DS/obese rats and their lean homozygous littermate (DahlS.Z‐*Lepr* ^*+*^ */Lepr* ^*+*^, or DS/lean) controls were provided with drinking water containing 0.50% L‐arginine‐HCl or 0.85% L‐alanine (isonitrogenous control) from 13 to 17 weeks of age. L‐Arginine supplementation markedly alleviated hypertension without affecting cardiac injury in MetS rats. It also attenuated the increase in ROS production apparent in cardiac mitochondria isolated from MetS rats as well as suppressed the associated upregulation of Nox4 mRNA and protein in the heart. Furthermore, L‐arginine reversed the decrease in the size of cardiac mitochondria as well as changes in the expression of DRP1 and OPA1 proteins apparent in the L‐alanine–treated MetS rat heart. Cardiac arginase II gene expression and arginase activity were increased by L‐arginine treatment in MetS rats but not CONT rats. L‐Arginine supplementation thus ameliorated hypertension and cardiac mitochondrial abnormalities in MetS rats, with the lack of a cardioprotective effect possibly being due to increased arginase activity.

## 1 INTRODUCTION

Metabolic syndrome (MetS) is determined by metabolic risk factors linked to obesity and insulin resistance and is characterized by hypertension, glucose intolerance, and dyslipidemia as well as a consequent increased susceptibility to the development of both cardiovascular disease and type 2 diabetes. Left ventricular (LV) hypertrophy and diastolic dysfunction are more common in hypertensive individuals with MetS than in those without it, in part as a result of increased cardiac inflammation and fibrosis (Sciarretta et al., 2007).

In addition to serving as a building block for protein synthesis, L‐arginine is a physiological substrate for nitric oxide (NO) production and thereby contributes to intracellular signal transduction (Kohli et al., 2014). It is also a substrate of arginase, which is activated pathologically as a result of oxidative stress and inflammation (Caldwell et al., 2018). Indeed, increased arginase activity has been associated with immune dysfunction and cancer in addition to cardiovascular pathologies including hypertension, atherosclerosis, cardiac ischemia, and reperfusion injury (3). Administration of L‐arginine was previously shown to reduce body fat mass and to improve glucose and lipid metabolism as well as insulin sensitivity in Zucker diabetic obese rats and rats with diet‐induced obesity (Fu et al., 2005; Jobgen et al., 2009).

Dynamic changes in mitochondrial morphology related to mitochondrial fusion and fission (mitochondrial dynamics) have recently been observed in association with changes in biological energy supply and demand as well as with pathological conditions, suggesting the possibility of a link between MetS and abnormal mitochondrial dynamics and function (Díaz‐Juárez & Suarez, 2017). Administration of L‐arginine ameliorated mitochondrial dysfunction in the brain cortex of rats with streptozotocin‐induced diabetes (Ortiz et al., 2013). In addition, a study of mice haploinsufficient for optic atrophy 1 (OPA1), which is required for mitochondrial fusion, suggested that such fusion protects against hypertension by suppressing the production of reactive oxygen species (ROS) in blood vessels (Robert et al., 2021). Moreover, treatment of rats with an NO synthase (NOS) inhibitor (L‐NAME) was found to downregulate and upregulate the expression of proteins required for mitochondrial fusion (OPA1) or fission (dynamin‐related protein 1 [DRP1]), respectively, in the aorta (Miller et al., 2013). These observations suggest an important relation among mitochondrial morphology and function, oxidative stress, and L‐arginine–dependent NO production. However, the effects of L‐arginine on cardiac mitochondrial ROS production and dynamics in metabolic diseases have remained unclear.

We have previously established an animal model of MetS, the DahlS.Z‐*Lepr* ^*fa*^/*Lepr* ^*fa*^ (DS/obese) rat, which is the result of a cross between Dahl salt‐sensitive (DS) rats and Zucker rats harboring a missense mutation in the leptin receptor gene (*Lepr*) (Hattori et al., 2011). DS/obese rats develop salt‐sensitive hypertension as well as LV diastolic dysfunction, hypertrophy, and fibrosis, with these alterations being associated with increased LV oxidative stress and inflammation (Murase et al., 2012). We have now examined the effects of L‐arginine supplementation on cardiac pathology, mitochondrial ROS production and dynamics, and metabolism in DS/obese rats.

## 2 MATERIALS AND METHODS

### 2.1 Animals and experimental protocols

The study was approved by the Animal Experiment Committee of Nagoya University Graduate School of Medicine (Daiko district, approval nos. D210008‐001, D220003‐001, and D230004‐002). Male inbred DS/obese and DahlS.Z‐*Lepr* ^+^/*Lepr* ^+^ (DS/lean) rats at 12 weeks of age were supplied by Japan SLC, Inc. (Hamamatsu, Japan) and were maintained and studied according to guidelines of Nagoya University Graduate School of Medicine and Guide for the Care and Use of Laboratory Animals (U.S. National Institutes of Health publication no. 85–23, revised 2011). After 1 week of acclimatization to a purified diet (D12450K; Research Diets, New Brunswick, NJ) with carbohydrate at 70 kcal%, fat at 10 kcal%, and casein at 20 kcal%, DS/obese rats were administered the diet supplemented with either 0.50% L‐arginine‐HCl (WK01804625; Fujifilm Wako Pure Chemical, Osaka, Japan) or 0.85% L‐alanine (WK01401045; Fujifilm Wako Pure Chemical) as an isonitrogenous control via drinking water from 13 to 17 weeks of age (*n* = 12 for both MetS + Arg and MetS + Ala groups). They were compared with similarly treated DS/lean littermate controls (*n* = 12 for both CONT + Ala and CONT + Arg groups). All rats had free access to the diet and supplemented tap water throughout the experimental period. Body weight and food and water intake were assessed weekly. Systolic blood pressure (SBP) was also measured on a weekly basis in conscious animals by tail‐cuff plethysmography (BP‐98AL; Softron, Tokyo, Japan), as previously detailed (Takatsu et al., 2013). At 17 weeks of age, the rats underwent an insulin tolerance test (ITT) (Takeshita et al., 2015) as well as transthoracic echocardiography and cardiac catheterization and were then killed by injection of a lethal dose of sodium pentobarbital (50 mg/kg, intraperitoneal). Blood glucose concentration was measured with the use of a glucose analyzer (Glutest Neo Super; Sanwa Kagaku Kenkyusho, Nagoya, Japan), and blood glucose responses during the ITT (0, 15, 30, 60, and 120 min) was calculated as the area under the curve (AUC) with the trapezoidal method. The heart, kidneys, and visceral (epididymal and retroperitoneal) and subcutaneous (inguinal) fat tissue were rapidly collected and weighed. LV tissue was dissected from the heart for analysis. The kidneys were cut vertically in the center to prepare specimens for histological examination, whereas the remaining tissue was subjected to mRNA analysis.

### 2.2 Echocardiography and cardiac catheterization

Rats at 17 weeks of age were subjected to anesthesia by injection of ketamine and xyalzine (50 and 10 mg/kg, respectively, intraperitoneal) followed by transthoracic echocardiography and cardiac catheterization, as described previously (Kato et al., 2008; Nagata et al., 2002). M‐mode echocardiography was conducted with a 12.5‐MHz transducer (Xario SSA‐660A; Toshiba Medical Systems, Tochigi, Japan), and LV end‐diastolic (LVDd) and end‐systolic (LVDs) dimensions, interventricular septum thickness (IVST), and LV posterior wall thickness (LVPWT) were determined. LV fractional shortening (LVFS), relative wall thickness (RWT), and LV mass were derived by the following formulas: LVFS (%) = [(LVDd – LVDs)/LVDd] × 100; RWT = (IVST + LVPWT)/LVDd; and LV mass (g) = (1.04 × [(IVST + LVDd + LVPWT)^3^ – LVDd^3^] × 0.8) + 0.14. LV ejection fraction (LVEF) was determined by the Pombo formula. For assessment of LV diastolic function, Doppler‐derived indices were obtained by recording the LV inflow velocity pattern with pulsed‐wave Doppler echocardiography. Peak flow velocities during rapid filling (E) and atrial contraction (A), deceleration time (DcT), and isovolumic relaxation time (IRT) were determined. After completion of echocardiography, a 2F micromanometer‐tipped catheter (SPR‐320NR; Millar Instruments, Houston, TX) calibrated relative to atmospheric pressure was positioned in the left ventricle via the right carotid artery (Kato et al., 2008). LV end‐diastolic pressure (LVEDP) was determined from digitized tracings of LV pressure and the electrocardiogram. The time constant of isovolumic relaxation (tau) was determined according to the derivative method of Raff and Glantz, as previously described (Nagata et al., 1995).

### 2.3 Histology and immunohistochemistry

LV or kidney tissue was fixed for 48 h with 4% paraformaldehyde in phosphate‐buffered saline, embedded in paraffin, and processed for histological analysis, as described previously (Kato et al., 2008; Nagata et al., 2002). Transverse sections of the left ventricle (thickness, 3 μm) and the kidney (thickness, 2 μm) were subjected to hematoxylin–eosin (H&E), Azan‐Mallory, or periodic acid–Schiff (PAS) staining as well as to immunohistochemical staining with antibodies to the monocyte–macrophage marker CD68 (clone ED‐1, diluted 1:100; MAB1435; Chemicon, Temecula, CA), as described previously (Miyachi et al., 2009). The glomerulosclerosis index (GSI) was determined from 50 glomeruli in PAS‐stained sections of each rat as a semiquantitative score ranging from 0 to 4, where 0 represents normal glomeruli, 1 indicates ≤25% involvement, 2 reflects 26%–50%, 3 corresponds to 51%–75%, and 4 denotes >75% sclerosis (Aoyama et al., 2020). The tubulointerstitial injury score (TIS) was evaluated in 10 fields per Azan‐Mallory–stained section for each rat, with the score ranging from 0 to 4 (0, normal interstitium; 1, ≤25% of area injured; 2, 26%–50% of area injured; 3, 51%–75% of area injured; and 4, >75% of area injured) (Aoyama et al., 2020). CD68‐positive cells were counted in 20 glomeruli for each rat as previously described (Aoyama et al., 2020). All image analysis was conducted with NIH Scion Image software (Scion, Frederick, MD) in a blinded manner with respect to the experimental status of the animals.

### 2.4 Assay of superoxide production

Nicotinamide adenine dinucleotide phosphate (NADPH)–dependent superoxide production by homogenates prepared from frozen LV tissue was assessed with the use of an assay based on lucigenin‐enhanced chemiluminescence, as previously described (Nagata et al., 2006). The chemiluminescence signal was recorded every minute for 10 min with a spectrophotometric microplate reader (WALLAC 1420 ARVO MX/Light; PerkinElmer, Waltham, MA), and the corresponding background counts were subtracted from the experimental values. Lucigenin chemiluminescence was quantified as relative light units (RLU) per minute per milligram of protein. Superoxide production in tissue sections was assessed by staining with dihydroethidium (Invitrogen, Carlsbad, CA), as described (Uchinaka et al., 2018). Dihydroethidium is rapidly oxidized by superoxide, resulting in the production of fluorescent ethidium, and the sections were examined with a fluorescence microscope equipped with a 585‐nm long‐pass filter. The average dihydroethidium fluorescence intensity values were calculated with NIH ImageJ software (Murase et al., 2012).

### 2.5 Analysis of mitochondrial ROS production

LV tissue was homogenized in an ice‐cold solution comprising 300 mM sucrose, 5 mM *N*‐Tris(hydroxymethyl)methyl‐2‐aminoethanesulfonic acid (TES), and 0.2 mM EGTA at a pH of 7.2. The homogenate was centrifuged at 800 × *g* for 5 min at 4°C, and the resulting supernatant was centrifuged further at 8300 × *g* for 10 min at 4°C. The resulting pellet was suspended in ice‐cold buffer, and the protein concentration of this mitochondrial fraction was determined (Samniang et al., 2016). ROS production by the isolated mitochondria was evaluated with the use of 2′,7′‐dichlorodihydrofluorescein diacetate (D399; Thermo Fisher Scientific, Waltham, MA). The mitochondria were incubated for 25 min at room temperature with the H_2_DCF‐DA dye at 2 μM, and the ROS level was quantified by measurement of fluorescence (excitation and emission at 485 and 535 nm, respectively) with a microplate reader (Infinite200PRO M Plex, Tecan).

### 2.6 Analysis of mitochondrial morphology by transmission electron microscopy (TEM)

Sample preparation for TEM was as previously described (Hammerschmidt et al., 2019). LV tissue was shredded into 1‐mm square pieces, fixed with 25% glutaraldehyde (TAAB Laboratories Equipment, Aldermaston, England), exposed to 4% osmium tetroxide (Nisshin‐EM, Tokyo, Japan), dehydrated with a series of ethanol solutions of increasing concentration, and embedded in epoxy resin for preparation of ultrathin sections with an ultramicrotome. The sections were collected on TEM grids and visualized with a TEM (JEM‐1400Plus; JEOL, Tokyo, Japan) operating at an acceleration voltage of 100 kV. The area of individual mitochondria was quantified by manual delineation of mitochondrial outlines in TEM images. The aspect ratio of individual mitochondria, reflecting elongation or roundness, was calculated as the major axis/minor axis length ratio. Mitochondrial roundness was also assessed on the basis of geometric descriptors. Circularity (values closer to 1 suggesting a more circular shape, and those deviating from 1 indicating elongation) was calculated as: (4π × area)/(perimeter^2^). The form factor, a dimensionless shape descriptor, was calculated as the inverse of circularity, providing insight into mitochondrial contour irregularity (smaller values indicating a more complex shape, and larger values a rounder shape). Finally, the total number of mitochondria within a defined area was determined systematically to provide a measure of mitochondrial density. The imaging magnification for the TEM analysis was set at 5000× and all data were analyzed with ImageJ (NIH, Bethesda, MD) software (Murase et al., 2012).

### 2.7 Assays for nitrogen oxides, L‐arginine, and arginase

Rats were deprived of food overnight, blood was collected from the right carotid artery and centrifuged at 1400 × *g* for 10 min at 4°C, and the serum supernatant was maintained at −80°C before analysis. The concentration of nitrogen oxides (NOx) in the serum was determined with a Nitrate/Nitrite Colorimetric Assay Kit (CAY780001; Cayman Chemical, Ann Arbor, MI). The reaction reagent was mixed with serum that had been depleted of protein with the use of a 10‐kDa spin filter in a Centrifugal Filter Unit (UCF501024; Millipore, Burlington, MA), and the absorbance of each sample was measured at 540 nm with a spectrophotometric microplate reader (Infinite200PRO M Plex; Tecan, Kawasaki, Japan). In addition, the concentration of L‐arginine in the serum was quantified with an enzyme‐linked immunosorbent assay kit (CEB938GE; Cloud‐Clone, Wuhan, China). The samples were incubated for 20 min at room temperature before measurement of absorbance at 450 nm with the microplate reader. For assay of arginase activity in LV tissue, total protein was isolated from the tissue and quantified as described previously (Hattori et al., 2014). In brief, LV tissue was homogenized on ice in a lysis buffer containing 0.1% Triton X‐100, 25 mM Tris–HCl (pH 7.5), and 1 mM MnSO_4_. The lysate was centrifuged at 15,000 × *g* for 2 min at 4°C, and the supernatant was assayed for protein content and then incubated at 57.5°C for 10 min. Equal amounts of protein were then assayed for arginase activity with a QuantiChrom Arginase Assay Kit (BAS031026; BioAssay Systems, Hayward, CA). The samples were incubated for 60 min at room temperature before measurement of absorbance at 430 nm with the microplate reader.

### 2.8 RT and real‐time PCR analysis

Total RNA was isolated from LV and kidney tissue as described previously (Chomczynski & Sacchi, 2006). In brief, frozen tissue samples were crushed in liquid nitrogen with the use of a chilled mortar and then homogenized in RNAiso Plus (9109; Takara, Shiga, Japan). A one‐fifth volume of chloroform (Fujifilm Wako Pure Chemical) was then added to the homogenate, and the mixture was agitated for 30 s with a vortex mixer and then centrifuged at 15,000 × *g* for 30 min at 4°C. The upper aqueous phase was transferred to another tube, to which 0.8× volume of isopropanol (Fujifilm Wako Pure Chemical) was added and the resulting mixture was again agitated for 30 s before centrifugation at 15,000 × *g* for 10 min at 4°C. The RNA pellet was washed with 75% ethanol (Fujifilm Wako Pure Chemical), air‐dried to remove residual ethanol, and reconstituted in RNase‐free water (Takara). The concentration and purity of the RNA were assessed by measurement of absorbance at 260 and 280 nm with a spectrophotometer (DU730; Beckman Coulter, Tokyo, Japan). Reverse transcription (RT) was performed with 2‐μg portions of the RNA and with the use of a PrimeScript RT Reagent Kit (RR037; Takara). Real‐time polymerase chain reaction (PCR) analysis was performed with the resulting cDNA and with the use of SYBR Mix Ex Taq II (RR820; Takara) and a Thermal Cycler Dice Real Time System II (Takara) (Matsuura et al., 2015). The PCR primer sequences are provided in Table 1. The amount of each target mRNA was normalized by that of glyceraldehyde‐3‐phosphate dehydrogenase (GAPDH) mRNA as an internal control.

**TABLE 1:** PCR primers for quantitative RT‐PCR analysis.

Target	Sequence	GenBank accession no.
SOD2	Forward 5’‐TGGAGAACCCAAAGGAGAGTTG‐3’	NM_017051.2
	Reverse 5’‐TATTGAAGCCAAGCCAGCCC‐3’	
Nox4	Forward 5’‐GCTTACCTTCGCGGATCACA‐3’	NM_053524.1
	Reverse 5’‐CAGCTACATGCACACCTGAGAA‐3’	
eNOS	Forward 5’‐AGGCAATCTTCGTTCAGCCA‐3’	NM_021838.2
	Reverse 5’‐CAGTGATCTCCACGTTGGCA‐3’	
iNOS	Forward 5’‐TGTGCTAATGCGGAAGGTCA‐3’	NM_012611.3
	Reverse 5’‐GTTCCATGCAGACAACCTTGG‐3’	
nNOS	Forward 5’‐ACATGCTGCTGGAGATCGG‐3’	NM_052799.2
	Reverse 5’‐CCATCTTCTTGGCTACTTCCTCC‐3’	
Arginase I	Forward 5’‐CTGCTGTGGTAGCAGAGACC‐3’	NM_017134.3
	Reverse 5’‐TGCTTCCAATTGCCATACTGTG‐3’	
Arginase II	Forward 5’‐AGAGGCTTTCTATGTTGGGATGC‐3’	NM_019168.2
	Reverse 5’‐ACTAACCACCTCAGCCAGTTCC‐3’	
ODC	Forward 5’‐TGTGAGGAGACAGCATTCAGAG‐3’	NM_001302083.1
	Reverse 5’‐GGTTCTCGATGTGCCTACAGA‐3’	
OAT	Forward 5’‐TGGTGGCTTATATCCCGTGTC‐3’	NM_022521.3
	Reverse 5’‐GTGCTCGCCTGGTTTAATGG‐3’	
ANP	Forward 5′‐GGCTCCTTCTCCATCACCAA‐3’	NM_012612.2
	Reverse 5′‐AGGTGGTCTAGCAGGTTCTTG‐3’	
BNP	Forward 5′‐CAATCCACGATGCAGAAGCTG‐3’	NM_031545.1
	Reverse 5′‐GGCGCTGTCTTGAGACCTAA‐3’	
Collagen type I	Forward 5’‐GTACATCAGCCCAAACCCCA‐3’	NM_053304.1
	Reverse 5’‐TCGCTTCCATACTCGACTGG‐3’	
Collagen type III	Forward 5’‐TGTGTGATGATGAGCCACTAGAC‐3’	NM_032085.1
	Reverse 5’‐GGAATGACAGGAGCAGGTGTA‐3’	
TGF‐β1	Forward 5’‐ACCAACTACTGCTTCAGCTCC‐3’	NM_021578.2
	Reverse 5’‐AGACAGAAGTTGGCATGGTAGC‐3’	
CTGF	Forward 5’‐GCTGCCTACCGACTGGAAG‐3’	NM_022266.2
	Reverse 5’‐CGCTCCACTCTGTGGTCTG‐3’	
MCP‐1	Forward 5’‐TATGCAGGTCTCTGTCACGC‐3’	nm_031530.1
	Reverse 5’‐GGCATTAACTGCATCTGGCTG‐3’	
TNF‐α	Forward 5’‐ATCGGTCCCAACAAGGAGGA‐3’	NM_012675.3
	Reverse 5’‐TCCGCTTGGTGGTTTGCTAC‐3’	
COX‐2	Forward 5’‐CCAACCTCTCCTACTACACCAG‐3’	NM_017232.4
	Reverse 5’‐GAAGTTCCTTATTTCCTTTCACACC‐3’	
p22^phox^	Forward 5’‐GCGGTGTGGACAGAAGTACC‐3’	NM_024160.2
	Reverse 5’‐CAGGCACGGACAGCAGTAA‐3’	
gp91^phox^	Forward 5’‐CACTTCACACGGCCATTCAC‐3’	NM_023965.1
	Reverse 5’‐TATGGGTCCGAAGTCCCGA‐3’	
Angiotensinogen	Forward 5’‐AGGTTTGTGCAGGCTGTGAC‐3’	NM_134432.2
	Reverse 5’‐GAAGCCTCTCATCTTCCCTTGG‐3’	
Renin	Forward 5’‐CCAAGATGTGGTAACTGTGGGTG‐3’	NM_012642.4
	Reverse 5’‐AGGGATGACTCCATCAACAGCC‐3’	
ACE	Forward 5’‐ACTATGCCAAGGTGCTGTTTGC‐3’	NM_012544.1
	Reverse 5’‐TCCTGTGTCTGAGAAGCCATCTTG‐3’	
AT_1A_	Forward 5’‐TCTCTCAGCTCTGCCACATTCC‐3’	NM_030985.4
	Reverse 5’‐TCGAAATCCACTTGACCTGGTG‐3’	
Collagen type IV	Forward 5’‐ATTCCTTTGTGATGCACACCAG‐3’	NM_001135009.1
	Reverse 5’‐AAGCTGTAAGCATTCGCGTAGTA‐3’	
GAPDH	Forward 5’‐GGCACAGTCAAGGCTGAGAATG‐3’	NM_017008.4
	Reverse 5’‐ATGGTGGTGAAGACGCCAGTA‐3’	

### 2.9 Immunoblot analysis

Total protein was extracted from LV tissue and quantified as previously described (Hattori et al., 2014). In brief, LV tissue was homogenized on ice in a lysis buffer containing a protease inhibitor cocktail (cOmplete EDTA‐free; Roche, Mannheim, Germany) and 1 mM sodium orthovanadate as an inhibitor of protein tyrosine phosphatases. The homogenate was centrifuged at 20,000 × *g* for 10 min at 4°C, and the supernatant was assayed for protein content. Equal amounts of the total protein samples were fractionated by SDS‐polyacrylamide gel electrophoresis, and the resulting protein bands were transferred to a polyvinylidene difluoride membrane (Xu et al., 2005). The membrane was exposed to mouse monoclonal antibodies to NADPH oxidase 4 (Nox4) (1:2000 dilution; 67,681‐1‐Ig; Proteintech, Tokyo, Japan) or with rabbit monoclonal antibodies to DRP1 (1:1000; 8570S; Cell Signaling Technology, Danvers, MA), to OPA1 (1:1000; 80471S; Cell Signaling Technology), or to GAPDH (1:1000; 2118S; Cell Signaling Technology). It was then exposed to horseradish peroxidase–conjugated horse antibodies to mouse immunoglobulin G (1:3000; 7076S; Cell Signaling Technology) or goat antibodies to rabbit immunoglobulin G (1:3000; 7074P2; Cell Signaling Technology). Immune complexes were detected and quantified as previously described (Hattori et al., 2014).

### 2.10 Biochemical analysis

Blood collected from the right carotid artery of rats that had been deprived of food overnight was centrifuged at 1400 × *g* for 10 min at 4°C, and the resulting serum supernatants were stored at −80°C until analysis. The levels of glucose, total cholesterol, low–density lipoprotein (LDL) cholesterol, high–density lipoprotein (HDL) cholesterol, triglyceride, free fatty acids, urea nitrogen, and creatinine in serum were measured by routine enzymatic assays. 24‐h creatinine clearance rate (24‐h CCr) was calculated using the standard formula UV/P, where U is the urinary creatinine concentration, V is the 24‐h urine volume and P is the serum creatinine concentration.

### 2.11 Statistical analysis

Data are presented as means ± SD. The independent (main effects) or interactive influence of genotype and L‐arginine on the investigated parameters in the four groups of rats at 17 weeks of age was evaluated by two‐way factorial analysis of variance (ANOVA). If the interaction was significant, intergroup comparisons were performed with the Bonferroni‐Dunn posthoc test; if the interaction was not significant, the main effects (genotype and L‐arginine) were simply interpreted. Time courses of body weight, food and water intake, and SBP were compared among groups by two‐way repeated‐measures ANOVA. A *p* value of <0.05 was considered statistically significant for ANOVA, while a *p* value of <0.0083 was for the Bonferroni‐Dunn posthoc test.

## 3 RESULTS

### 3.1 Influences of genotype and L‐arginine

All of the study data were subjected to two‐way factorial ANOVA in order to assess the influences of rat genotype and L‐arginine supplementation and the possible interaction of these factors (Table S1).

### 3.2 Metabolic and physiological parameters

**FIGURE 1:**
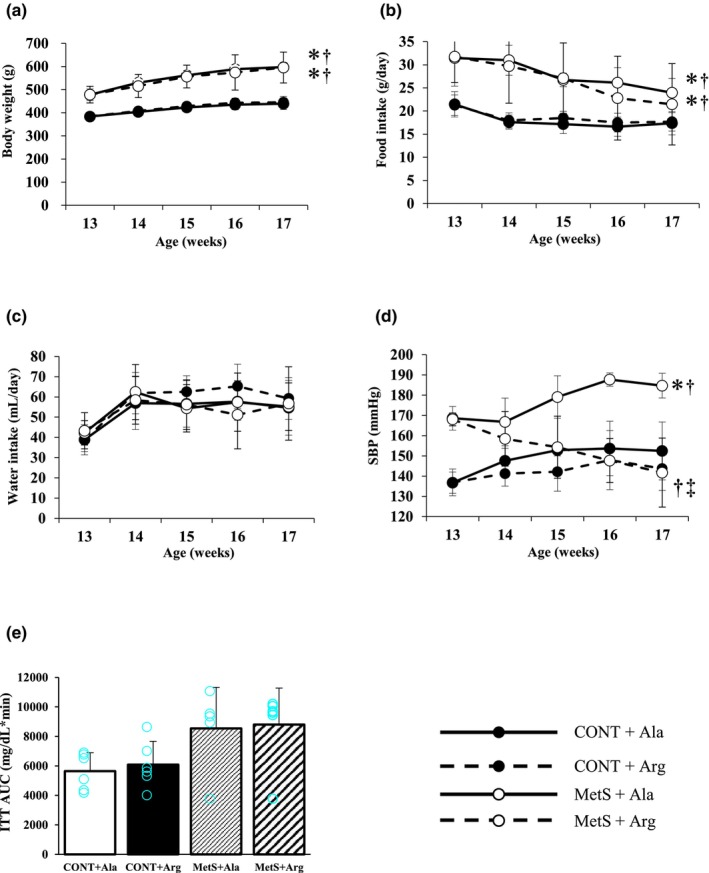
Time courses of body weight (a), food intake (b), water intake (c), and SBP (d) as well as an ITT AUC (e) in rats of the four experimental groups. Data are means ± SD. In (a–c), *n* = 11, 12, 11, and 11; in (d), *n* = 9, 10, 7, and 8; and in (e), *n* = 6, 6, 5, and 6 for the CONT + Ala, CONT + Arg, MetS + Ala, and MetS + Arg groups, respectively. *p < 0.0083 vs. CONT + Ala, †*p* < 0.0083 vs. CONT + Arg, ‡*p* < 0.0083 vs. MetS + Ala (the Bonferroni‐Dunn posthoc test).

We first examined the effects of L‐arginine supplementation on various metabolic and physiological parameters. Whereas body weight (Figure 1a, Table 2) and food intake (Figure 1b, Table 2) were increased in the MetS + Ala group compared with the CONT + Ala group, supplementation with L‐arginine did not affect these parameters in either rat strain. Furthermore, water intake did not differ among the four experimental groups (Figure 1c, Table 2). L‐Arginine supplementation ameliorated the increase in SBP apparent in the MetS + Ala group (Figure 1d, Table 2), whereas ITT data revealed that insulin sensitivity was impaired in MetS rats compared with CONT rats in a manner insensitive to L‐Arginine supplementation (Figure 1e, Table S1). At 17 weeks of age, MetS rats manifested increases in heart, LV, and both visceral (epididymal and retroperitoneal) and subcutaneous (inguinal) fat weight compared with littermate controls, but none of these parameters, except heart weight, was affected by L‐arginine supplementation (Table 2).

**TABLE 2:** Physiological parameters for rats of the four experimental groups at 17 weeks of age.

Parameter	CONT + ala	CONT + Arg	MetS + ala	MetS + Arg
Body weight (g)	439.3 ± 25.2	445.4 ± 25.4	597.5 ± 18.5	595.6 ± 66.7
Food intake (g/day)	17.3 ± 2.51	17.7 ± 1.99	24.0 ± 3.12	21.5 ± 8.79
Water intake (mL/day)	54.7 ± 13.54	59.2 ± 10.34	55.2 ± 11.74	56.8 ± 18.13
SBP (mmHg)	152.4 ± 14.29	143.7 ± 10.75	184.7 ± 6.11	141.8 ± 17.05
Heart weight/tibial length (mg/mm)	33.96 ± 2.45	36.58 ± 2.80	43.11 ± 4.16	45.38 ± 6.66
LV weight/tibial length (mg/mm)	25.04 ± 2.08	26.87 ± 2.43	32.20 ± 3.68	33.72 ± 6.01
Epididymal fat weight/tibial length (mg/mm)	182.32 ± 35.41	197.92 ± 30.96	351.02 ± 24.50	350.12 ± 46.11
Retroperitoneal fat weight/tibial length (mg/mm)	182.97 ± 38.04	190.21 ± 39.13	649.66 ± 51.90	642.86 ± 101.88
Inguinal fat weight/tibial length (mg/mm)	352.44 ± 82.76	349.07 ± 103.50	2208.82 ± 357.94	2334.11 ± 497.47
Tibia length (mm)	38.97 ± 0.69	40.37 ± 2.54	37.47 ± 0.74	36.49 ± 1.71

*Note*: Data are means ± SD (*n* = 11, 12, 10, and 11 for CONT + Ala, CONT + Arg, MetS + Ala, and SMetS + Arg groups, respectively).

### 3.3 Cardiac morphology and function

Echocardiography and cardiac catheterization were performed to characterize the effects of L‐arginine supplementation on LV morphology and function in rats at 17 weeks of age (Table 3). IVST, LVPWT, RWT, and LV mass were all increased in MetS rats compared with CONT rats, indicative of LV hypertrophy, as were both LVFS and LVEF. The E/A ratio was reduced, whereas IRT, DcT, and tau were increased, in MetS rats, indicative of impaired LV relaxation, and the LVEDP/LVDd ratio, an index of LV diastolic stiffness, was also increased. However, none of these cardiac parameters, except LVPWT or RWT, was influenced by L‐arginine supplementation.

**TABLE 3:** Cardiac morphology and function for rats of the four experimental groups at 17 weeks of age.

Parameter	CONT + ala	CONT + Arg	MetS + ala	MetS + Arg
IVST (mm)	1.85 ± 0.13	1.91 ± 0.13	2.19 ± 0.24	2.12 ± 0.22
LVDd (mm)	7.48 ± 0.63	7.89 ± 0.64	7.39 ± 0.48	7.66 ± 0.18
LVPWT (mm)	1.90 ± 0.13	1.87 ± 0.14	2.13 ± 0.11	2.05 ± 0.16
LVFS (%)	40.47 ± 6.58	41.08 ± 3.74	51.94 ± 11.09	49.50 ± 7.33
LVEF (%)	78.02 ± 6.36	79.07 ± 3.90	86.79 ± 7.33	85.21 ± 5.35
RWT	0.51 ± 0.07	0.49 ± 0.07	0.59 ± 0.08	0.55 ± 0.05
LV mass (mg)	1039.95 ± 89.16	1114.26 ± 104.25	1250.68 ± 72.91	1262.60 ± 170.55
E/A	2.17 ± 0.27	2.13 ± 0.29	1.69 ± 0.36	1.69 ± 0.21
IRT (ms)	26.60 ± 5.80	25.03 ± 5.51	38.88 ± 7.67	38.40 ± 8.37
DcT (ms)	38.18 ± 5.98	40.33 ± 12.26	53.28 ± 6.32	52.08 ± 7.53
Tau (ms)	0.019 ± 0.0009	0.019 ± 0.002	0.027 ± 0.004	0.028 ± 0.005
LVEDP/LVDd (mmHg/mm)	0.44 ± 0.09	0.59 ± 0.09	0.87 ± 0.16	0.82 ± 0.06

*Note*: Data are means ± SD (*n* = 11, 12, 10, and 10 [echocardiography]; *n* = 5, 5, 5, and 5 [cardiac catheterization] for CONT + Ala, CONT + Arg, MetS + Ala, and MetS + Arg groups, respectively).

### 3.4 Cardiac pathology

We also investigated the effects of L‐arginine supplementation on LV remodeling and injury. The cross‐sectional area of LV cardiomyocytes was increased in MetS rats compared with CONT rats (Figure S1a,b), and hemodynamic overload increased expression of the atrial natriuretic factor (ANP) and brain natriuretic factor (BNP) genes in the heart of these animals (Figure S1c,d). However, none of these parameters was affected by L‐arginine supplementation. The extents of perivascular and interstitial fibrosis in the LV myocardium, as revealed by Azan‐Mallory staining, were increased in MetS rats compared with CONT rats (Figure S2a–d). Expression of the collagen types I and III, transforming growth factor–β1 (TGF‐β1), and connective tissue growth factor (CTGF) genes correlates with cardiac fibrosis and was also increased in the heart of MetS rats (Figure S2e–h). However, again, L‐arginine supplementation did not affect any of these fibrosis‐related parameters.

### 3.5 Cardiac inflammation, oxidative stress, and RAS‐related gene expression

Immunohistochemical staining for CD68, a marker for monocytes and macrophages, showed that the extent of infiltration of these cells in the LV myocardium was increased in MetS rats compared with CONT rats (Figure S3a,b). The expression of monocyte chemoattractant protein–1 (MCP‐1), tumor necrosis factor–α (TNF‐α), and cyclooxygenase‐2 (COX‐2) genes was also upregulated in the left ventricle of MetS rats (Figure S3c–e). However, none of these markers of LV inflammation was affected by L‐arginine supplementation. Superoxide production in cardiac tissue sections, as revealed by dihydroethidium staining (Figure S3f, g), as well as NADPH oxidase (Nox) activity in LV homogenates (Figure S3h) and the expression of genes for the p22^phox^ and gp91^phox^ (Nox2) membrane components of Nox in LV tissue (Figure S3i,j) were all increased in MetS rats compared with CONT rats. None of these parameters related to LV oxidative stress was influenced by L‐arginine supplementation. Probing of the renin‐angiotensin system (RAS) revealed that the amounts of mRNAs for angiotensinogen, renin, angiotensin converting enzyme (ACE), and the angiotensin II type 1A receptor (AT_1A_) were increased in LV tissue of MetS rats relative to that of CONT rats (Figure S4). However, supplementation with L‐arginine had no effect on the expression of these genes.

### 3.6 Cardiac mitochondrial ROS production

**FIGURE 2:**
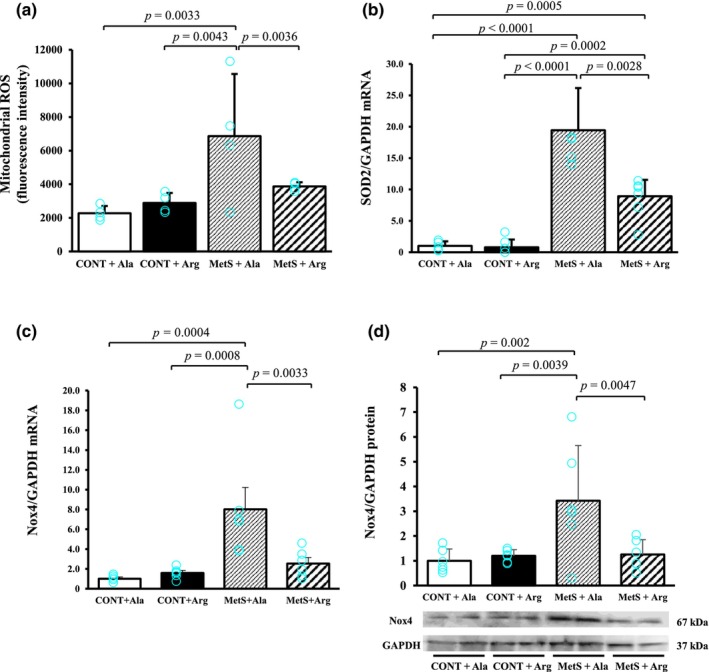
LV mitochondrial function in 17‐week‐old rats. (a) ROS production revealed by H_2_DCF‐DA staining for isolated LV mitochondria. (b, c) Relative SOD2, and Nox4 mRNA abundance, respectively, in LV tissue. (d) Representative immunoblot analysis and densitometric quantification of the abundance of Nox4 and GAPDH (loading control) in LV tissue. All quantitative data are means ± SD, with *n* = 4, 4, 4, and 4 (A); *n* = 5, 5, 5, and 6 (b); *n* = 5, 5, 5, and 5 (c); and *n* = 6, 6, 6, and 6 (d) for CONT + Ala, CONT + Arg, MetS + Ala, and MetS + Arg groups, respectively.

To explore the effects of L‐arginine supplementation on cardiac mitochondrial oxidative stress, we measured ROS production in mitochondria isolated from LV tissue. The production of ROS was increased in MetS + Ala rats compared with CONT +Ala rats, and this effect was attenuated in MetS + Arg rats (Figure 2a). In contrast, the amounts of superoxide dismutase 2 (SOD2) mRNA as well as Nox4 mRNA and protein were increased in the MetS + Ala group compared with the CONT + Ala group, and these effects were attenuated by L‐arginine supplementation (Figure 2b–d).

### 3.7 Cardiac mitochondrial dynamics

**FIGURE 3:**
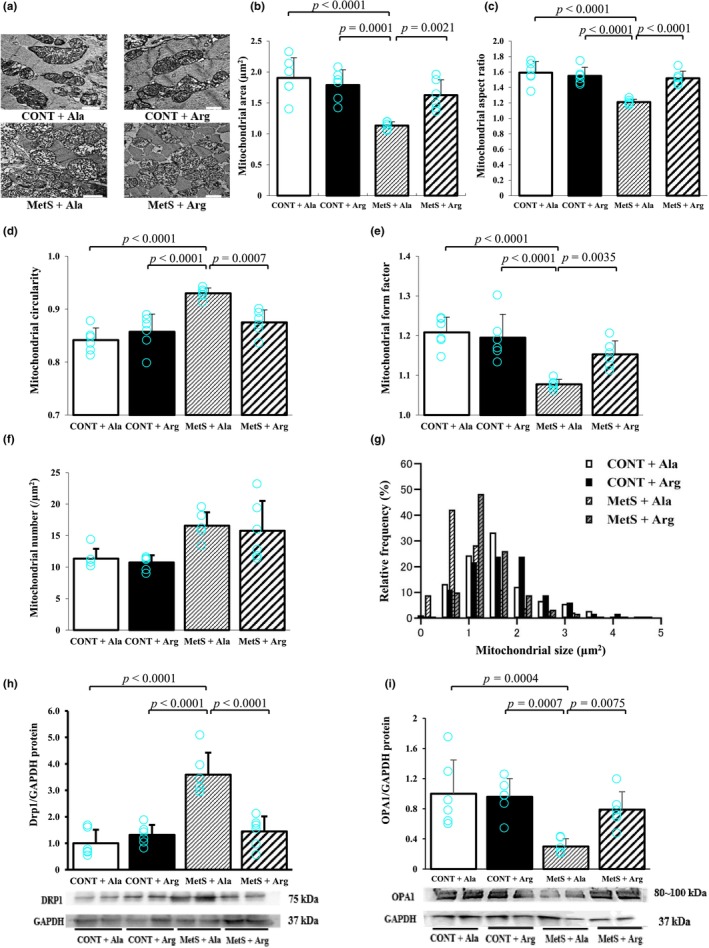
LV mitochondrial dynamics in rats at 17 weeks of age. (a) TEM of transverse sections of LV tissue showing mitochondria. Scale bars, 1.0 μm. (b–f) Mitochondrial area (b), aspect ratio (c), circularity (d), form factor (e), and density (f) determined from images as in (a). (g) Relative frequency of mitochondrial size based on area, also determined from images as in (a). (h, i) Representative immunoblot analysis and densitometric quantification of DRP1 (h), OPA1 (i), and GAPDH in LV tissue. All quantitative data are means ± SD, with *n* = 6 in (b) to (i) for each group.

TEM of LV tissue sections revealed that mitochondrial area, aspect ratio (length/width ratio), and form factor were reduced, whereas mitochondrial circularity was increased, in MetS + Ala rats compared with CONT + Ala rats (Figure 3a–e), and all of these effects were attenuated in MetS + Arg rats. The number of mitochondria was increased in MetS rats compared with CONT rats, but it was unaffected by L‐arginine supplementation (Figure 3f). The peak of the frequency distribution for mitochondrial size in the MetS + Arg group was shifted to the right compared with that in the MetS + Ala group (Figure 3g). Immunoblot analysis revealed that the amount of the mitochondrial fission protein DRP1 was increased, whereas that of the mitochondrial fusion protein OPA1 was reduced, in LV tissue of MetS + Ala rats compared with that of CONT + Ala rats, and that these effects were attenuated in MetS + Arg rats (Figure 3h,i).

### 3.8 L‐arginine metabolism

**FIGURE 4:**
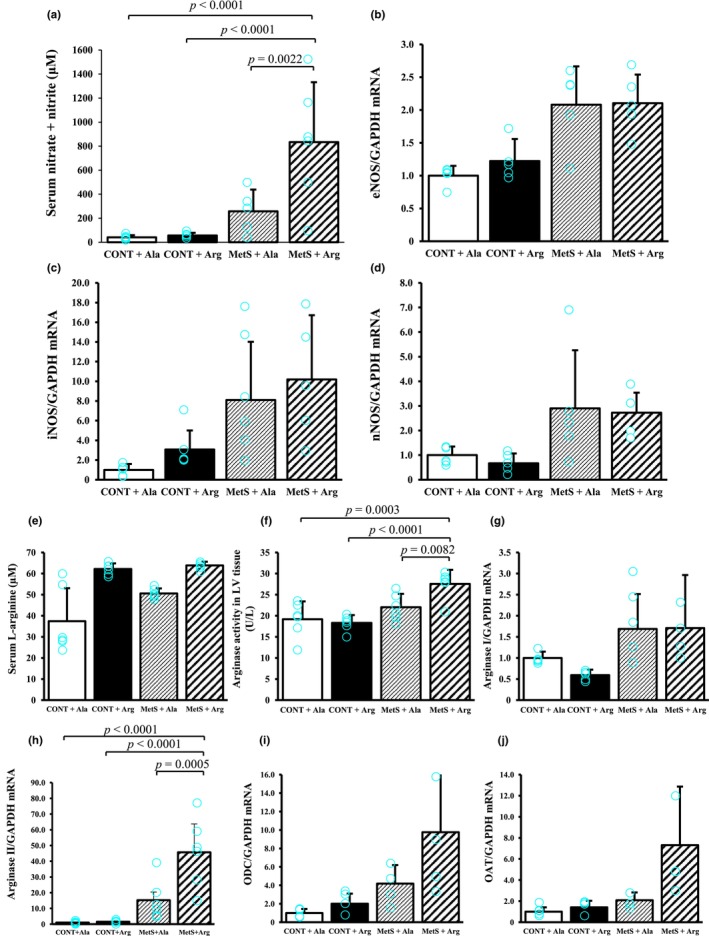
L‐Arginine metabolism in 17‐week‐old rats. (a) Serum nitrate + nitrite (NOx) concentration. (b–d) Relative eNOS, iNOS, and nNOS mRNA abundance, respectively, in LV tissue. (e) Serum L‐arginine concentration. (f) Arginase activity in LV tissue. (g–j) Relative arginase I, arginase II, ODC, and OAT mRNA abundance, respectively, in LV tissue. All quantitative data are means ± SD, with *n* = 6, 6, 6, and 6 (A, E, F, H); *n* = 5, 5, 5, and 5 (b, d); *n* = 5, 5, 6, and 6 (C); *n* = 5, 5, 5, and 6 (g); and *n* = 4, 4, 4, and 4 (i, j) for CONT + Ala, CONT + Arg, MetS + Ala, and MetS + Arg groups, respectively.

We investigated the effects of L‐arginine supplementation on NOS and arginase pathways in the heart. The concentration of NOx in serum did not differ between MetS + Ala rats and CONT + Ala or CONT + Arg rats, but it was increased in MetS + Arg rats compared with the other three groups (Figure 4a). The expression of genes for the endothelial (eNOS), inducible (iNOS), and neuronal (nNOS) forms of NOS in the left ventricle was upregulated in MetS rats compared with CONT rats, but it was not affected by L‐arginine supplementation (Figure 4b–d). The concentration of L‐arginine in serum was higher in MetS rats than in CONT rats and was increased by L‐arginine supplementation, although the interaction was not significant (Figure 4e). Arginase activity in LV tissue was similar in the MetS + Ala and CONT + Ala groups and was increased in the MetS + Arg group (Figure 4f). The amount of arginase I mRNA in the heart was increased in MetS rats compared with CONT rats, but it was not influenced by L‐arginine supplementation (Figure 4g). In contrast, the amount of arginase II mRNA in the heart did not differ between the MetS + Ala and CONT + Ala groups and was increased in the MetS + Arg group (Figure 4h). The amount of ornithine decarboxylase (ODC) mRNA in LV tissue was increased in MetS rats compared with CONT rats, but it was not influenced by L‐arginine supplementation (Figure 4i). The amount of ornithine aminotransferase (OAT) mRNA in LV tissue was increased in MetS rats compared with CONT rats, and it was influenced by L‐arginine supplementation (Figure 4j).

### 3.9 Renal pathology

We also examined the effects of L‐arginine supplementation on renal damage and gene expression. The GSI (Figure S5a,b), the number of CD68‐positive cells in glomeruli (Figure S5c,d), and renal expression of MCP‐1, TNF‐α, and COX‐2 genes (Figure S5e–g) were all increased in MetS rats compared with CONT rats. However, L‐arginine supplementation did not affect any of these parameters of glomerular injury and inflammation. The tubulointerstitial injury score (TIS) (Figure S6a,b) was higher in MetS rats than in CONT rats, as were the amounts of collagen types I, III, and IV as well as TGF‐β1 and CTGF mRNAs in the kidney (Figure S6c–g). However, again, none of these parameters related to peritubular fibrosis was affected by L‐arginine supplementation.

### 3.10 Biochemical analysis

Assessment of the effects of L‐arginine supplementation on biochemical parameters revealed that serum levels of glucose and free fatty acids were similar in the four experimental groups (TableS2). The serum concentrations of total cholesterol, low density lipoprotein (LDL)–cholesterol, high density lipoprotein (HDL)–cholesterol, triglyceride, urea nitrogen, and creatinine and urinary protein levels were increased, whereas the 24‐h creatinine clearance rate (24‐h CCr) was decreased, in MetS rats compared with CONT rats (TableS2), but L‐arginine supplementation did not influence these parameters related to lipid metabolism or renal function.

## 4 DISCUSSION

We have here shown that L‐arginine supplementation ameliorated hypertension and cardiac mitochondrial abnormalities, but not cardiac and renal injury, in MetS rats. Although such supplementation did not influence body weight, food intake, or water intake, it resulted in an increase in the serum concentration of L‐arginine. The serum concentration of NOx as well as arginase activity in the left ventricle were increased by L‐arginine supplementation specifically in MetS rats.

### 4.1 The L‐arginine paradox

We here found that L‐arginine supplementation markedly attenuated hypertension in MetS rats. L‐Arginine serves as a physiological substrate for NO production by NOS (Gokce, 2004). NOS exists as three isozymes (eNOS, nNOS, and iNOS), with eNOS and nNOS being constitutively expressed in the cardiovascular system (Gambardella et al., 2020). In addition, eNOS has been localized to caveolae of the plasma membrane and is able to translocate to and from the cytoplasm (Koo et al., 2018; Pandey et al., 2014). We found that the serum concentration of L‐arginine ranged from ~40 to 50 μM in CONT + Ala rats and MetS + Ala rats. It was lower than the reported concentration of L‐arginine in cells (100 to 800 μM) but was much higher than the Michaelis constant (*K*_m_) of eNOS for L‐arginine (3 μM) (Wu & Morris, 1998), suggesting that eNOS should be saturated with L‐arginine under physiological conditions. Although the *K*_m_ of arginase for L‐arginine (2 mM) is ~1000 times that of eNOS, the maximal velocity (*V*_max_) of arginase is also ~1000 times that of eNOS, resulting in approximately equal activities of the two enzymes for L‐arginine metabolism. Given the high affinity of eNOS for L‐arginine, it is unlikely that the intracellular concentration of L‐arginine could fall to a level that leaves the enzyme in a substrate‐deficient state. Nevertheless, L‐arginine supplementation in both animals and humans was shown to enhance NO‐mediated biological effects in vascular endothelial cells (the L‐arginine paradox) (Caldwell et al., 2018). In these cells, L‐arginine is converted by eNOS to NO and citrulline. Elevated NO levels promote vasodilation and thereby reduce peripheral vascular resistance and blood pressure as well as inhibit cardiomyocyte hypertrophy and proliferation and cardiac inflammation (Caldwell et al., 2018). These considerations together with the increased NOx concentration in serum suggest that the anti‐hypertensive effect of L‐arginine supplementation in MetS rats is attributable to increased production of NO by eNOS. The absence of an ameliorative effect on renal injury also suggests that the increased circulating levels of NO induced by L‐arginine supplementation are derived mostly from the vasculature, not from the kidneys.

### 4.2 Why does L‐arginine supplementation not ameliorate cardiac injury?

L‐Arginine supplementation substantially ameliorated hypertension but it did not attenuate cardiac injury in MetS rats. Arginase and NOS compete for their shared substrate L‐arginine. We found that arginase activity and expression of the arginase II gene were both increased in the heart of MetS rats by L‐arginine supplementation, possibly accounting for the lack of an effect on cardiac injury despite the anti‐hypertensive effect. This notion is consistent with previous studies indicating no cardiovascular benefit or an adverse effect of chronic L‐arginine supplementation (Lucas et al., 2014), likely as a result of counterregulatory effects of L‐arginine on arginase that reduce the substrate supply for eNOS. The increased oxidative stress and inflammation in the heart of MetS rats is also consistent with previous findings that increased arginase expression or activity results from intracellular signaling events including those triggered by ROS and inflammatory cytokines such as TNF‐α and interleukin‐6 (Hu et al., 2015; Huang et al., 2016). Arginase I is predominantly expressed in the liver and is a cytoplasmic protein that colocalizes with ODC. Arginase II is expressed in the cardiovascular system, including cardiomyocytes as well as vascular endothelial and smooth muscle cells, and it is predominantly but not exclusively a mitochondrial protein that colocalizes with OAT (Caldwell et al., 2018; Pernow & Jung, 2013). Arginase activity in the vascular wall and plasma was found to increase with age in obese rats (Peyton et al., 2018). In such rats, administration of L‐arginine or the arginase inhibitor *N* ^ω^‐hydroxy‐nor‐L‐arginine (nor‐NOHA) for 4 weeks starting at 8 weeks of age increased the plasma concentrations of L‐arginine and NOx, with both interventions reducing mean blood pressure (Peyton et al., 2018). In addition, increased expression of arginase II was detected in the aorta and myocardium of type 2 diabetic Goto‐Kakizaki rats compared with those of control (Wistar) rats (Grönros et al., 2011). However, it is important to note that arginase activity and expression are not always correlated. For example, whereas arginase activity was found to be increased in the left ventricle and lungs of spontaneously hypertensive rats, arginase I and II expression remained unchanged (Bagnost et al., 2009). Furthermore, both arginase activity and arginase I and II expression in the kidneys, liver, and brain of such rats were similar to those in control (Wistar Kyoto) rats (Bagnost et al., 2009). Although the reason for such discrepancy between arginase activity and isoform expression remains unclear, as mentioned above we found that the administration of L‐arginine resulted in increased arginase activity and arginase II gene expression in the heart of MetS rats. Overactive arginase is of pathological relevance to diabetes, hypertension, aging, and cancer. Arginase is integral to the urea circuit and is activated by ROS or inflammatory cytokines, contributing to an increase in L‐ornithine levels (3) that leads to increased polyamine and L‐proline production and promotion of cell growth, cell proliferation, and collagen synthesis (3). These processes are implicated in a pro‐inflammatory modulation of cardiac hypertrophy and fibrosis. Our results showing increased expression of ODC and OAT genes in the left ventricle of MetS rats are thus suggestive of activation of the arginase‐ornithine pathway, although the influence of L‐arginine supplementation on such expression in MetS rats was not statistically confirmed because of the lack of an interactive effect of genotype and L‐arginine.

### 4.3 Effects of L‐arginine on cardiac mitochondria in MetS

L‐Arginine supplementation attenuated the increase in ROS production by mitochondria and improved mitochondrial dynamics in the heart of MetS rats. Mitochondria are dynamic organelles that undergo cycles of fission and fusion that influence their morphology and distribution (Bereiter‐Hahn & Vöth, 1994). Mitochondrial dynamics are closely related to energy supply and demand. Under pathological conditions such as hyperglycemia, dyslipidemia, and obesity in MetS models, an increased energy supply induces mitochondrial fission and fragmentation as well as impairs mitochondrial respiration and ATP synthesis. Conversely, acute stress, cell proliferation (G_1_‐S phase), and food deprivation induce mitochondrial fusion and elongation as well as increase mitochondrial respiration (Liesa & Shirihai, 2013). The relation between mitochondrial function and ROS production is complex, with a loss of mitochondrial function having been found to either induce or suppress ROS production. Our findings are consistent with a previous result showing that exposure of cells to high glucose concentrations was found to promote mitochondrial fission, resulting in increased mitochondrial respiration and ROS production (Yu et al., 2006), with mitochondrial fission having therefore been suggested to be necessary for increased ROS production. ROS production is often associated with a loss of respiratory function and a decrease in mitochondrial membrane potential (ΔΨm) (depolarization). However, exposure of yeast cells maintained in glucose medium to moderate heat shock resulted in an increase in both ΔΨm (hyperpolarization) and mitochondrial ROS production (Pyatrikas et al., 2015). Although the underlying mechanisms remain unclear, these various observations suggest that mitochondrial function and dynamics are closely related.

Our present results suggest that exogenous L‐arginine inhibited mitochondrial fission and facilitated mitochondrial fusion in the heart of MetS rats, resulting in an amelioration of the abnormal cardiac mitochondrial dynamics apparent in these animals without an effect on mitochondrial number. The frequency distribution of mitochondrial area showed a rightward shift in the MetS + Arg group compared with the MetS + Ala group, indicative of an increase in mitochondrial size. NO production is thought to be related to mitochondrial function and dynamics. Mitochondrial function was found to be impaired in the brain cortex of streptozotocin‐induced diabetic rats, and L‐arginine administration improved mitochondrial function in association with a reduction in oxidative stress and increased NO production (7). Moreover, treatment of rats with the NOS inhibitor L‐NAME reduced the fusion and increased division of mitochondria in the aorta (9), and analysis of mitochondrial dynamics in vascular smooth muscle cells (mesenteric artery) isolated from OPA1 haploinsufficient mice after such L‐NAME treatment revealed enhanced mitochondrial fission (8). These findings suggest the importance of eNOS‐mediated NO production for mitochondrial formation. Our study now suggests that L‐arginine administration may protect against mitochondrial dysfunction in a manner dependent on the hypotensive effect of NO and restore the normal cycle of mitochondrial function and dynamics. However, we cannot exclude the possibility that L‐arginine acts directly on cardiac mitochondria as a nutritional factor.

Intracellular sources of ROS include Nox2 and xanthine oxidase in addition to mitochondria (electron transfer chain and Nox4), with these sources engaging in cross talk. Depletion of mitochondrial ROS by treatment of rats with a mitochondrially targeted antioxidant peptide attenuated angiotensin II–induced Nox4 expression, mitochondrial damage, mitochondrial biogenesis, p38 mitogen‐activated protein kinase (MAPK) phosphorylation, and apoptosis in cardiac myocytes (Dai et al., 2011). These effects were accompanied by amelioration of angiotensin II–induced cardiac hypertrophy, fibrosis, and diastolic dysfunction, despite the absence of a blood pressure–lowering effect. We found that, despite its alleviation of mitochondrial oxidative stress, L‐arginine supplementation did not reduce overall cellular oxidative stress in the heart of MetS rats. At least part of the protective effect of L‐arginine supplementation on cardiac mitochondria is likely attributable to the anti‐hypertensive effect of L‐arginine and its inhibition of mitochondrial fission and promotion of fusion. Nevertheless, the inability of such treatment to ameliorate cardiac injury, as reflected by cellular oxidative stress and inflammation, is suggestive of the involvement of increased arginase II activity. Whereas arginase I is thought to have an anti‐inflammatory function, arginase II is thought to have a pro‐inflammatory action (Li et al., 2022). In addition, arginase expression and activity are increased by ROS and inflammatory cytokines, and arginase itself has been shown to produce ROS (O_2_^−^) and reactive nitrogen species (ONOO^−^) and thereby to contribute to inflammation (Hara et al., 2020; Yao et al., 2017). ROS and inflammatory cytokines such as TNF‐α engage in cross talk (Blaser et al., 2016), suggesting that the activation of arginase may give rise to a vicious cycle involving ROS, reactive nitrogen species, and inflammatory cytokines. Both mitochondrial and cytoplasmic arginase II may contribute to the activation of Nox2 at the plasma membrane. L‐Arginine supplementation alleviated the increase in mitochondrial ROS production, resulting in an overall reduction in mitochondrial oxidative stress. SOD catalyzes the conversion of O_2_^−^ to H_2_O_2_ and exists as three isoforms (SOD1 to SOD3), with SOD2 being predominantly a mitochondrial Mn^2+^‐dependent enzyme. Depletion of mitochondrial SOD2 was found to increase Nox activity, whereas SOD2 overexpression attenuated Nox activation (Dikalova et al., 2010). The interplay between Nox and mitochondria is dependent on the mitochondrial O_2_^−^ level both in vitro and in vivo. Our results now suggest that downregulation of SOD2 gene expression in the left ventricle of MetS rats induced by L‐arginine supplementation may have contributed to the disparate findings regarding oxidative stress. The decrease in Nox4 expression in mitochondria coupled with the increase in arginase II (mitochondrial and cytoplasmic) gene expression in the heart of MetS rats induced by L‐arginine supplementation may have resulted in an overall lowering of mitochondrial oxidative stress. Whereas arginase I (cytoplasmic) gene expression remained unaffected by L‐arginine supplementation, the increase in arginase II gene expression may have acted to increase cellular ROS and Nox2 activity at the plasma membrane. However, the decrease in blood pressure induced by L‐arginine–derived NO may have acted to downregulate cellular ROS and Nox2 and Nox4 activities. These counteracting effects may explain the disparate results for L‐arginine supplementation between mitochondrial and cellular oxidative stress.

### 4.4 Study limitations

Our study lacks data on vascular endothelial function. Endothelial dysfunction is associated with cardiovascular risk factors including insulin resistance and may increase peripheral resistance via mechanisms that lead to increased constriction and vascular remodeling in resistance arteries, giving rise to the development of hypertension and other complications (Murray et al., 2021). A better understanding of the mechanisms underlying the anti‐hypertensive effect of L‐arginine supplementation may therefore be obtained by future studies with aortic rings ex vivo.

## 5 CONCLUSIONS

L‐Arginine supplementation substantially alleviated hypertension, but it did not ameliorate cardiac injury, in MetS rats. It also attenuated mitochondrial oxidative stress but not cellular oxidative stress in the heart of these animals. The effects of L‐arginine supplementation on cardiac pathology likely depend on the balance between NOS and arginase isoform expression. Our results raise the questions of whether the NOS‐mediated reduction in blood pressure and improvement of mitochondrial abnormalities are followed by the onset of arginase‐dependent cardiac remodeling, and whether the balance between NOS and arginase expression varies among different organs and will therefore give rise to diverse effects of L‐arginine supplementation. Future studies are warranted to characterize the molecular mechanisms responsible for the effects of L‐arginine supplementation on hypertension and cardiac pathology in MetS.

## 6 NEW & NOTEWORTHY

The effects of L‐arginine on cardiac mitochondrial ROS production and dynamics in metabolic diseases have remained unclear. L‐Arginine supplementation substantially alleviated hypertension, but it did not ameliorate cardiac injury, in metabolic syndrome rats. It also attenuated mitochondrial oxidative stress but not cellular oxidative stress in the heart of these animals. The effects of L‐arginine supplementation on cardiac pathology likely depend on the balance between nitric oxide synthase and arginase isoform expression.

## AUTHOR CONTRIBUTIONS

K.T.: performed experiments, analyzed data, prepared figures, and drafted, edited, and revised the manuscript. T.O., K.Y. and R.M.: performed experiments and analyzed data. N.O., A.K., M.K., and T.T.: performed experiments. K. Itakura: provided technical assistance for TEM and interpreted results. K. Ikeda and T.M.: critically revised the manuscript for intellectual content. K.N.: conceived, designed and supervised the research, obtained funding, interpreted results, edited and revised the manuscript. All authors approved the final version of the manuscript.

## FUNDING INFORMATION

This work was supported by a grant from the Ministry of Education, Culture, Sports, Science, and Technology of Japan (21 K08078 to K.N.) as well as by personal funds from Keiko Nagata, Tetsuro Nagata, Shigeki Nagata, and K.N.

## CONFLICT OF INTEREST STATEMENT

The authors declare no competing interests.

## Supporting information


Figure S1.

File S1.

Table S1.
Table S1.
Table S2.

## Data Availability

The data that support the results of this study are available from the corresponding author upon reasonable request.
