# Supplementary material for: L‐arginine ameliorates hypertension and cardiac mitochondrial abnormalities but not cardiac injury in male metabolic syndrome rats
Source: Physiol Rep. 2025 Feb 20;13(4):e70183. doi: 10.14814/phy2.70183 (PMC11842508; doi:10.14814/phy2.70183)
Supplement: Supplementary file 1 — Figure S1. [file PHY2-13-e70183-s003.pdf]

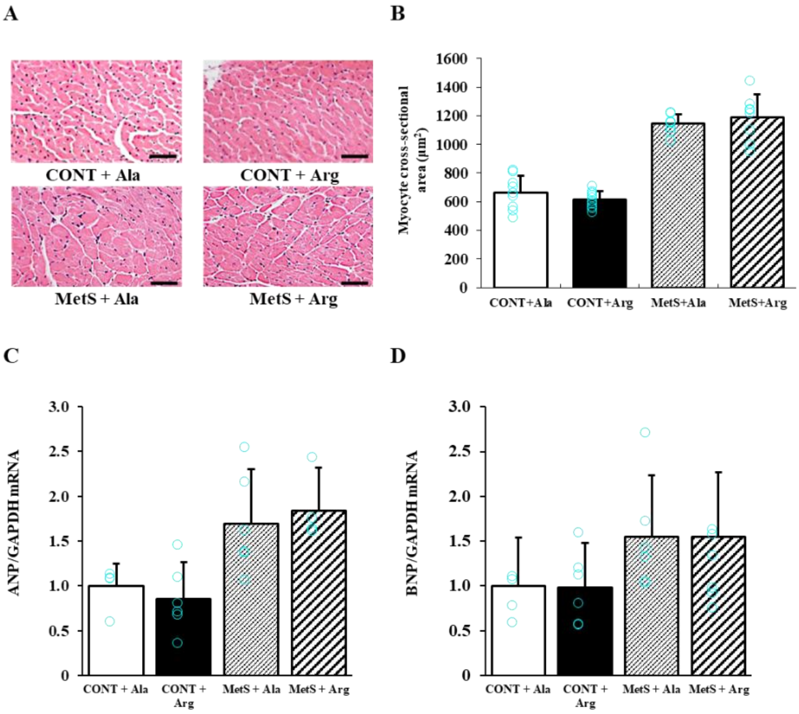

FIGURE S1 Cardiomyocyte hypertrophy in the left ventricle of rats from the four experimental groups at 17 weeks of age. (A) Representative H&E staining of transverse sections of the LV myocardium. Scale bars, 50 μm. (B) Cross-sectional area of LV myocytes as determined from sections similar to those in (A). (C, D) Quantitative RT-PCR analysis of relative ANP (C) and BNP (D) mRNA abundance in LV tissue. The amount of each mRNA was normalized by that of GAPDH mRNA and then expressed relative to the normalized value for the CONT + Ala group. All quantitative data are means ± SEM, with n = 9, 10, 8, and 8 in (B) and n = 6, 6, 6, and 6 in (C) and (D) for CONT + Ala, CONT + Arg, MetS + Ala, and MetS + Arg groups, respectively.

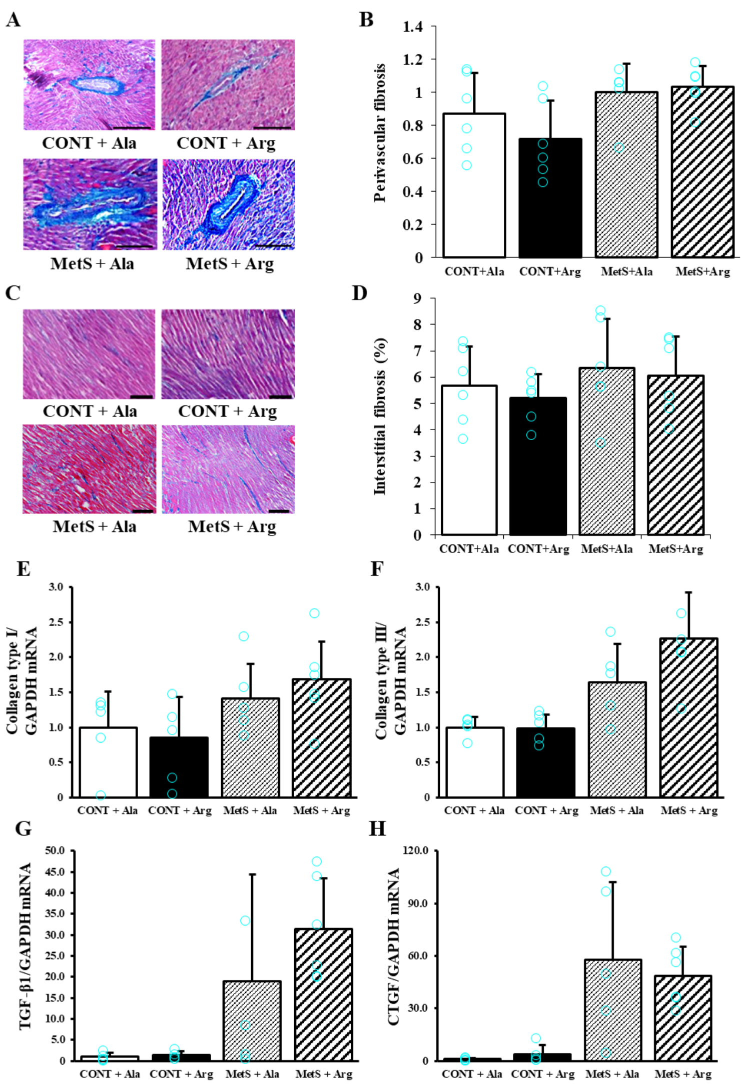

FIGURE S2 Fibrosis in the left ventricle of rats from the four experimental groups at 17 weeks of age. (A, C) Representative Azan-Mallory staining of fibrosis in perivascular (A) and interstitial (C) regions of the LV myocardium. Scale bars, 50 µm. (B, D) Quantitation of the relative extents of fibrosis determined from sections similar to those in (A) and (C), respectively. (E–H) Quantitative RT-PCR analysis of relative collagen types I and III, TGF-β1, and CTGF mRNA abundance, respectively, in LV tissue. The amount of each mRNA was normalized by that of GAPDH mRNA and then expressed relative to the normalized value for the CONT + Ala group. All quantitative data are means ± SEM, with *n* = 6, 6, 6, and 6 in (B) and (D) and *n* = 5, 5, 5, and 6 in (E) to (H) for CONT + Ala, CONT + Arg, MetS + Ala, and MetS + Arg groups, respectively.

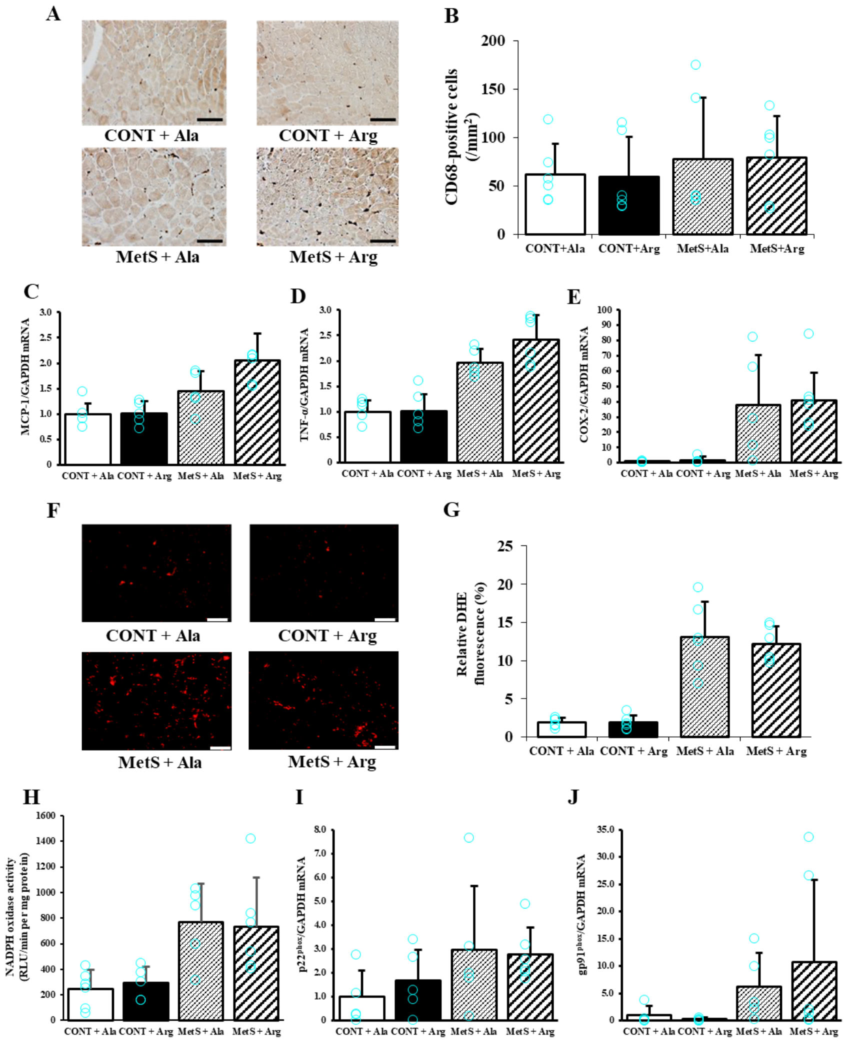

FIGURE S3 LV inflammation and oxidative stress in rats of the four experimental groups at 17 weeks of age. (A) Representative immunohistochemical staining of CD68 in the LV myocardium. Scale bars, 50 μm. (B) Quantitation of $CD68^{+}$ cell density determined from sections similar to those in (A). (C–E) Quantitative RT-PCR analysis of MCP-1, TNF-α, and COX-2 mRNA abundance, respectively, in LV tissue. The amount of each mRNA was normalized by that of GAPDH mRNA and then expressed relative to the normalized value for the CONT + Ala group. (F) Representative microscopic images of superoxide production in the LV myocardium as revealed by staining with dihydroethidium. Scale bars, 20 μm. (G) Dihydroethidium (DHE) fluorescence intensity as determined from sections similar to those in (F). (H) NADPH-dependent superoxide production in LV homogenates. Results are expressed as relative light units (RLU) per minute per milligram of protein. (I, J) Quantitative RT-PCR analysis of $p22^{phox}$ and $gp91^{phox}$ mRNA abundance, respectively, in LV tissue. All quantitative data are means ± SEM, with n = 9, 10, 8, and 8 in (B); n = 5, 5, 5, and 6 in (C) to (E), (H), (I), and (J); and n = 6, 6, 6, and 6 in (G) for CONT + Ala, CONT + Arg, MetS + Ala, and MetS + Arg groups, respectively.

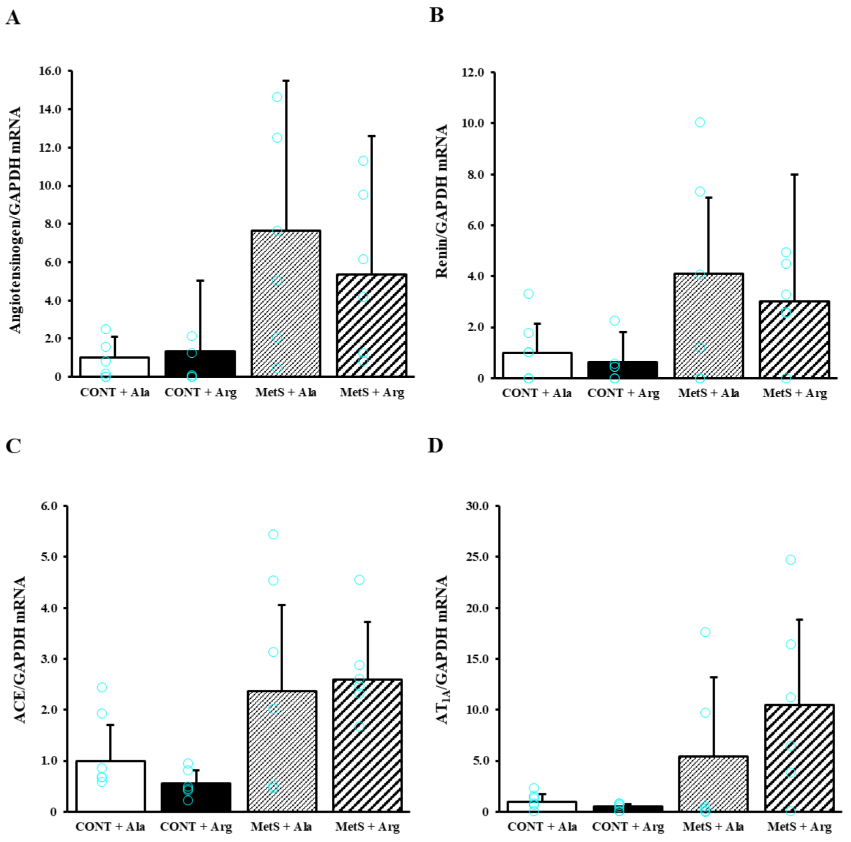

FIGURE S4 RAS-related gene expression in the left ventricle of rats from the four experimental groups at 17 weeks of age. The amounts of angiotensinogen (A), renin (B), ACE (C), and AT_{1A} (D) mRNAs were determined by quantitative RT-PCR analysis. The amount of each mRNA was normalized by that of GAPDH mRNA and then expressed relative to the normalized value for the CONT + Ala group. All quantitative data are means ± SEM, with *n* = 6 for each group.

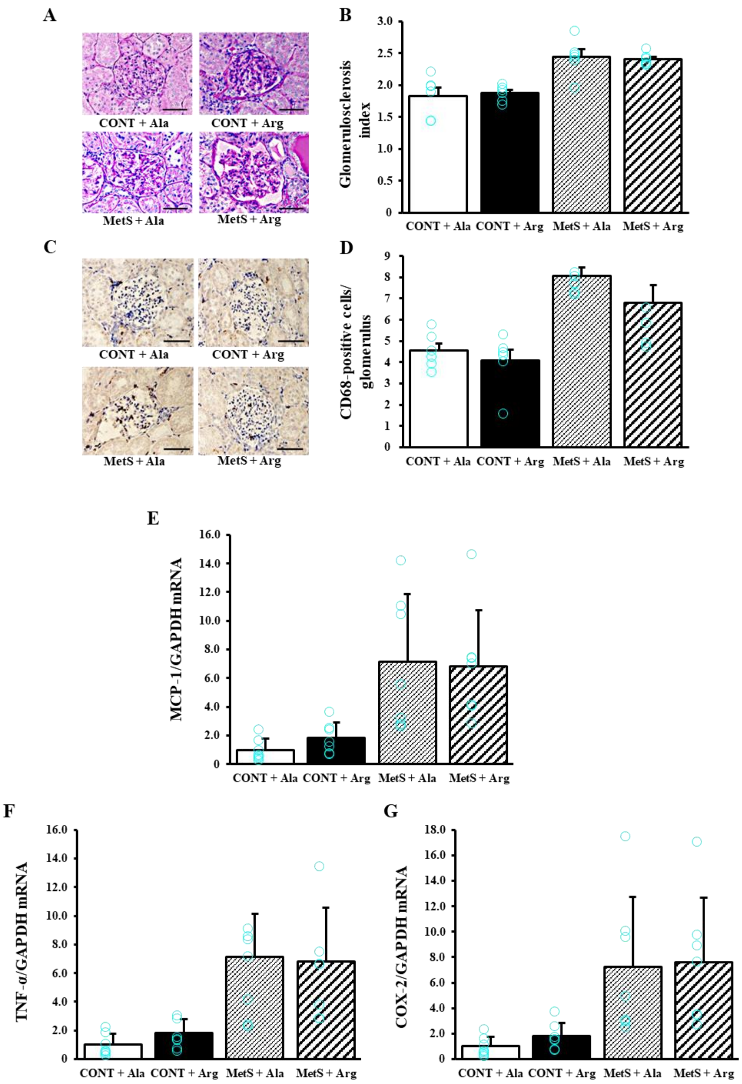

FIGURE S5 Renal histology and inflammation in rats of the four experimental groups at 17 weeks of age. (A) PAS staining of transverse sections of glomeruli. Scale bars, 20 µm. (B) The GSI (range of 0 to 4) as determined from sections similar to those in (A). (C) Representative immunohistochemical staining of CD68 in the kidney. Scale bars, 20 µm. (D) Number of nuclei for CD68-positive cells in each glomerulus as determined from sections similar to those in (C). (E–G) Quantitative RT-PCR analysis of relative MCP-1, TNF-α, and COX-2 mRNA abundance, respectively, in the kidney. The amount of each mRNA was normalized by that of GAPDH mRNA and then expressed relative to the normalized value for the CONT + Ala group. All quantitative data are means ± SEM, with n = 6 for each group in (B) and (D) and n = 7 for each group in (E) to (G).

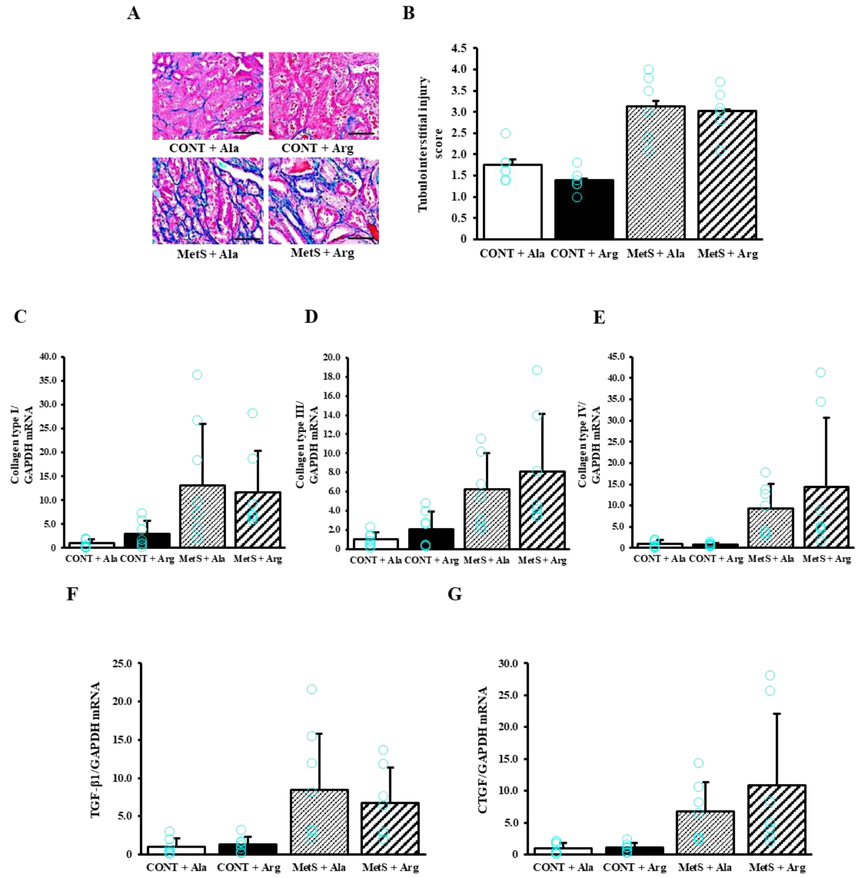

FIGURE S6 Renal fibrosis in rats of the four experimental groups at 17 weeks of age. (A) Representative Azan-Mallory staining of tubulointerstitial regions. Scale bars, 20 μm. (B) The TIS (range of 0 to 4) as determined from sections similar to those in (A). (C–G) Quantitative RT-PCR analysis of collagen types I, III, and IV as well as TGF-β1 and CTGF mRNA abundance, respectively, in the kidney. The amount of each mRNA was normalized by that of GAPDH mRNA and then expressed relative to the normalized value for the CONT + Ala group. All quantitative data are means ± SEM, with n = 6 for each group in (B) and n = 7 for each group in (C) to (G).
