# Supplementary material for: L‐arginine ameliorates hypertension and cardiac mitochondrial abnormalities but not cardiac injury in male metabolic syndrome rats
Source: Physiol Rep. 2025 Feb 20;13(4):e70183. doi: 10.14814/phy2.70183 (PMC11842508; doi:10.14814/phy2.70183)

Full unedited gels for Figure 2D

Nox4

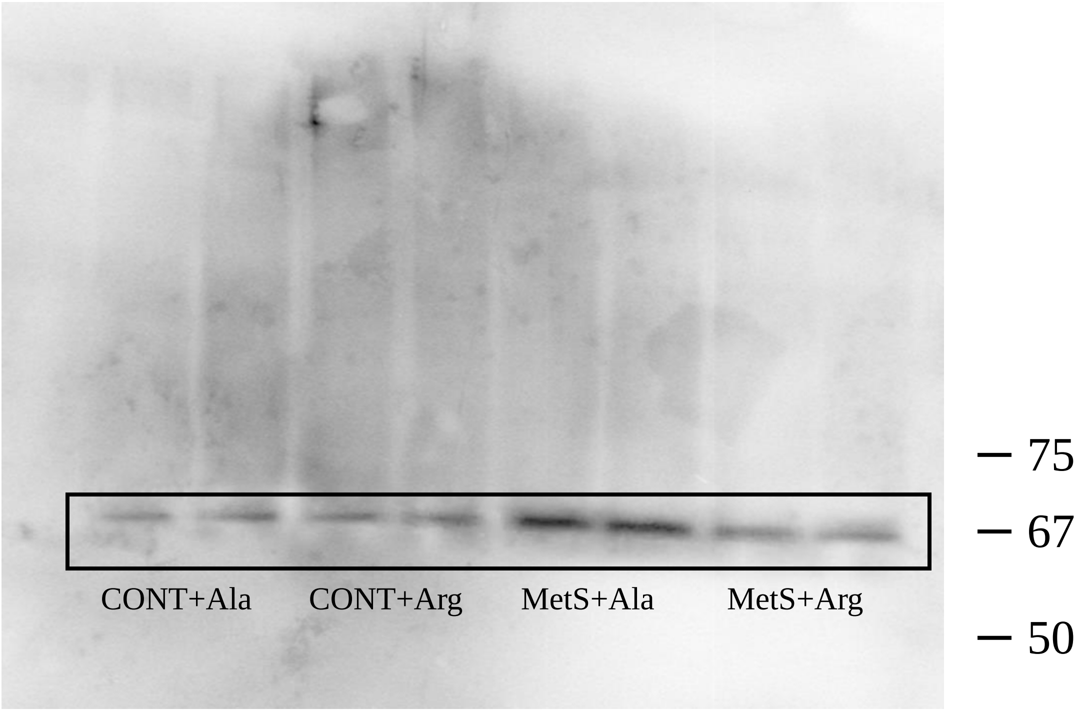

GAPDH

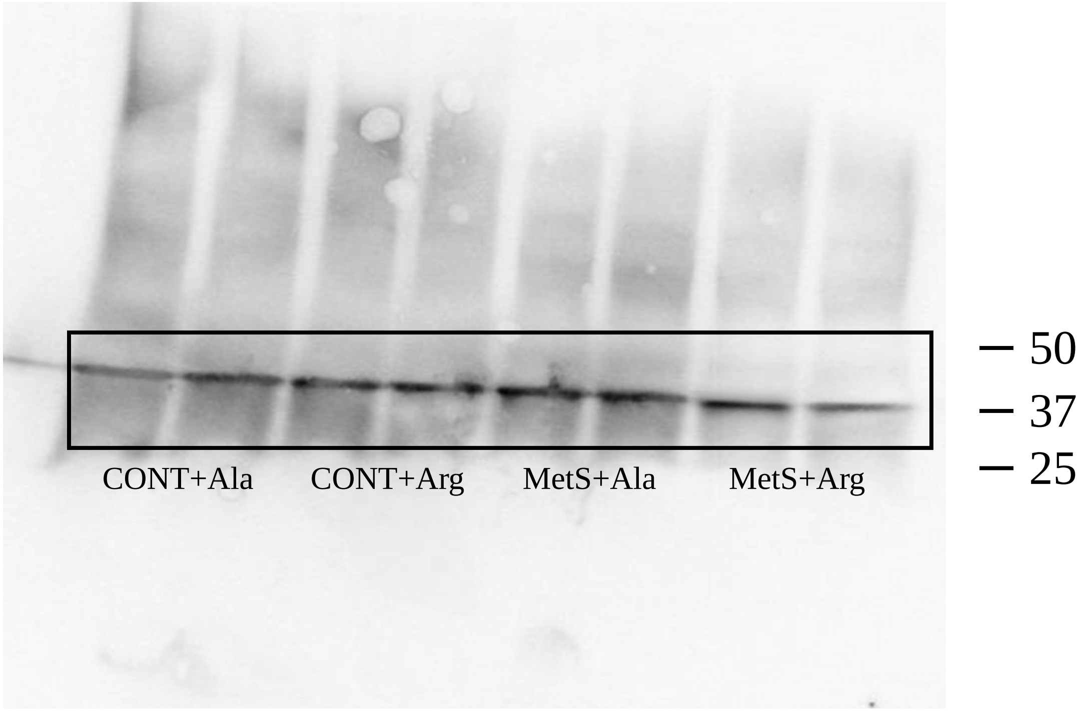

Full unedited gels for Figure 3H

DRP1

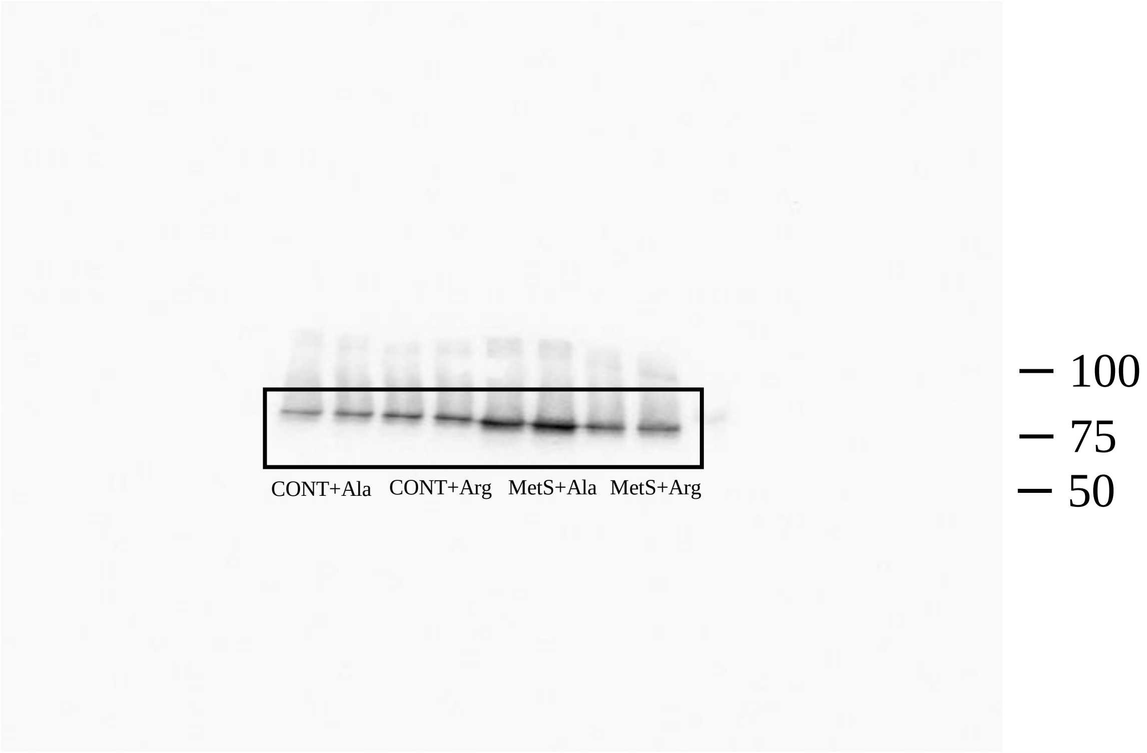

GAPDH

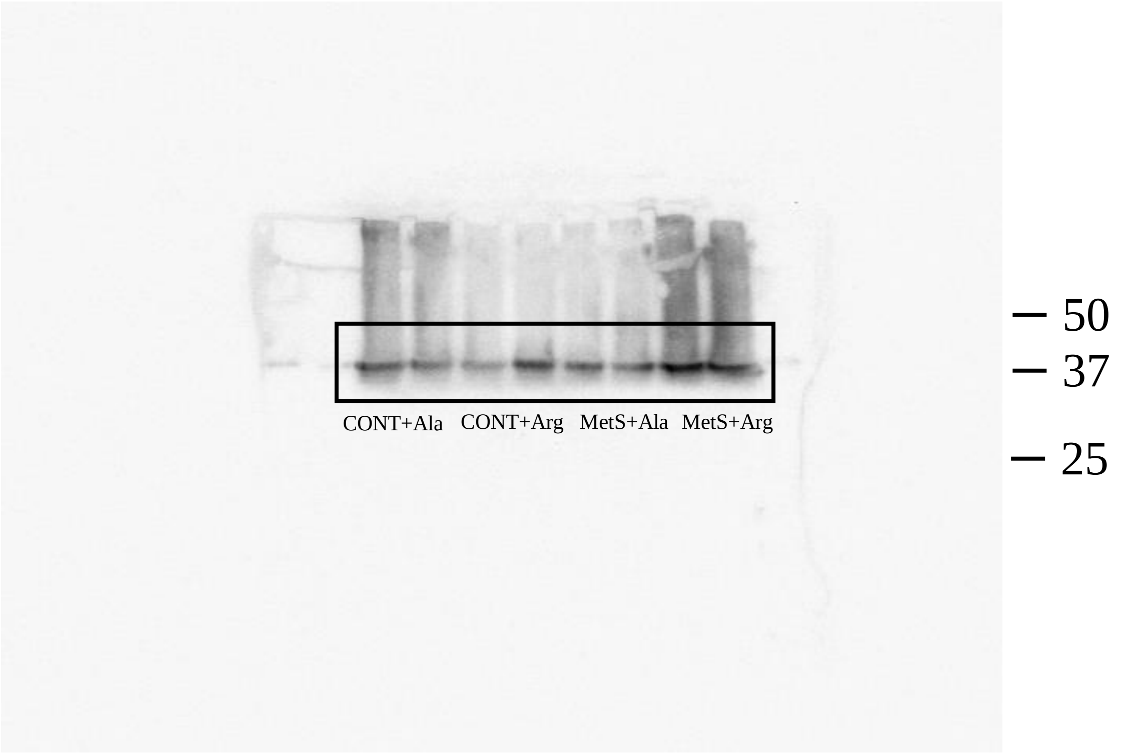

Full unedited gels for Figure 3I

OPA1

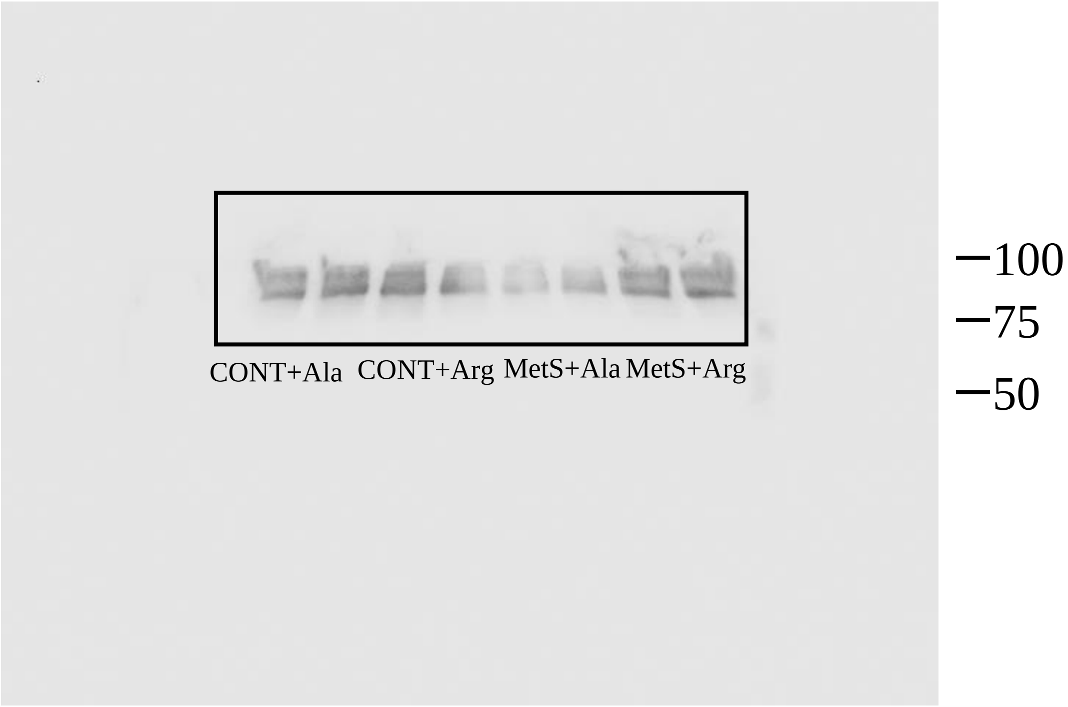

GAPDH

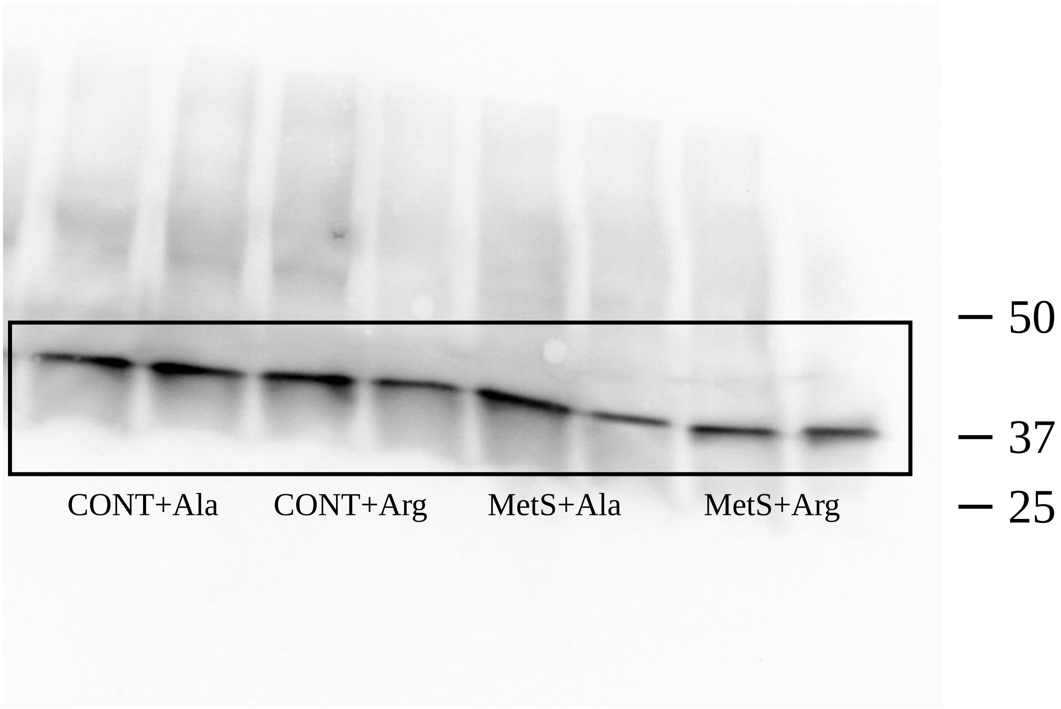

Supplement: Supplementary file 2 — File S1. [file PHY2-13-e70183-s002.pdf]
