# Supplementary material for: L‐arginine ameliorates hypertension and cardiac mitochondrial abnormalities but not cardiac injury in male metabolic syndrome rats
Source: Physiol Rep. 2025 Feb 20;13(4):e70183. doi: 10.14814/phy2.70183 (PMC11842508; doi:10.14814/phy2.70183)
Supplement: Supplementary file 3 — Table S1. Table S1. Table S2. [file PHY2-13-e70183-s001.docx]

**TABLE S1** Results of two-way factorial ANOVA for rat genotype and L-arginine supplementation in the four experimental groups (CONT + Ala, CONT + Arg, MetS + Ala, and MetS + Arg).

| **Parameter** | **Influence of genotype** | | **Influence of L-arg** | | **Interaction** | |
| --- | --- | --- | --- | --- | --- | --- |
|  | ***F* value** | ***P* value** | ***F* value** | ***P* value** | ***F* value** | ***P* value** |
| Body weight (g) | 139.980 | <0.0001 | 0.578 | 0.4532 | 2.239 | 0.1450 |
| Food intake (g/day) | 7.030 | 0.0125 | 1.735 | 0.1974 | 2.828 | 0.1021 |
| Water intake (mL/day) | 0.697 | 0.4103 | 0.427 | 0.5181 | 0.228 | 0.6363 |
| ITT AUC (mg/dL*min) | 10.451 | 0.0044 | 0.162 | 0.6915 | 0.010 | 0.9196 |
| SBP (mmHg) | 11.675 | 0.0018 | 33.958 | <0.0001 | 14.872 | 0.0006 |
| Heart weight/tibial length(mg/mm) | 47.343 | <0.0001 | 4.988 | 0.0331 | 0.001 | 0.9760 |
| LV weight/tibial length (mg/mm) | 30.343 | <0.0001 | 3.286 | 0.0799 | 0.003 | 0.9571 |
| Epididymal fat weight/tibial length (mg/mm) | 232.395 | <0.0001 | 0.143 | 0.7084 | 2.801 | 0.1046 |
| Retroperitoneal fat weight/tibial length (mg/mm) | 518.343 | <0.0001 | 0.034 | 0.8541 | 0.505 | 0.4826 |
| Inguinal fat weight/tibial length (mg/mm) | 372.368 | <0.0001 | 0.121 | 0.7300 | 0.114 | 0.7385 |
| Tibial length (mm) | 6.264 | 0.0165 | 0.037 | 0.8478 | 1.234 | 0.2732 |
| IVST (mm) | 19.280 | <0.0001 | 0.488 | 0.4890 | 1.698 | 0.2002 |
| LVDd (mm) | 1.682 | 0.2023 | 4.246 | 0.0460 | 0.539 | 0.4673 |
| LVPWT (mm) | 22.096 | <0.0001 | 4.450 | 0.0414 | 0.566 | 0.4564 |
| LVFS (%) | 21.233 | <0.0001 | 1.591 | 0.2147 | 0.434 | 0.5141 |
| LVEF (%) | 23.004 | <0.0001 | 1.204 | 0.2793 | 0.223 | 0.6397 |
| RWT | 13.319 | 0.0008 | 4.519 | 0.0399 | 0.365 | 0.5490 |
| LV mass (mg) | 14.477 | 0.0005 | 0.431 | 0.5152 | 2.820 | 0.1011 |
| E/A | 17.729 | 0.0001 | 0.218 | 0.6431 | 0.709 | 0.4049 |
| DcT (ms) | 9.972 | 0.0030 | 0.197 | 0.6596 | 3.950 | 0.0537 |
| IRT (ms) | 17.392 | 0.0002 | 1.281 | 0.2644 | 0.857 | 0.3601 |
| Tau (ms) | 30.578 | <0.0001 | 0.255 | 0.6207 | 0.311 | 0.5850 |
| LVEDP/LVDd  (mmHg/mm) | 44.167 | <0.0001 | 1.099 | 0.3101 | 3.984 | 0.0633 |
| LV myocyte cross-sectional area (µm^2^) | 221.945 | <0.0001 | 0.019 | 0.8918 | 1.100 | 0.3023 |
| ANP/GAPDH  mRNA in LV tissue | 44.450 | <0.0001 | 2.643 | 0.1107 | 0.447 | 0.5071 |
| BNP/GAPDH  mRNA in LV tissue | 10.661 | 0.0019 | 0.004 | 0.9529 | 0.002 | 0.9618 |
| Perivascular fibrosis  in LV tissue | 10.708 | 0.0038 | 0.209 | 0.6522 | 0.745 | 0.3983 |
| Interstitial fibrosis  in LV tissue (%) | 9.140 | 0.0067 | 0.273 | 0.6073 | 0.060 | 0.8093 |
| Collagen type I/GAPDH mRNA in LV tissue | 6.977 | 0.0171 | 0.010 | 0.9210 | 0.589 | 0.4534 |
| Collagen type III/GAPDH mRNA in LV tissue | 21.946 | 0.0002 | 2.239 | 0.1529 | 2.245 | 0.1524 |
| TGF-β1/GAPDH  mRNA in LV tissue | 13.719 | 0.0018 | 0.363 | 0.5551 | 0.305 | 0.5881 |
| CTGF/GAPDH  mRNA in LV tissue | 24.470 | 0.0001 | 0.099 | 0.7571 | 0.354 | 0.5598 |
| CD68-positive cells  in LV tissue (/mm^2^) | 74.742 | <0.0001 | 0.109 | 0.7434 | 0.109 | 0.7434 |
| MCP-1/GAPDH  mRNA in LV tissue | 16.176 | 0.0009 | 3.437 | 0.0812 | 3.350 | 0.0848 |
| TNF-α/GAPDH  mRNA in LV tissue | 52.642 | <0.0001 | 2.183 | 0.1579 | 1.655 | 0.2155 |
| COX-2/GAPDH  mRNA in LV tissue | 18.947 | 0.0004 | 0.127 | 0.7256 | 0.069 | 0.7958 |
| Relative DHE fluorescence (%) | 97.943 | <0.0001 | 0.175 | 0.6802 | 0.159 | 0.6939 |
| NADPH oxidase activity  (RLU min^–1^ mg^–1^ protein) | 18.095 | 0.0005 | 0.003 | 0.9559 | 0.115 | 0.7382 |
| p22^phox^/GAPDH mRNA  in LV tissue | 4.836 | 0.0420 | 0.072 | 0.7916 | 0.658 | 0.4284 |
| gp91^phox^/GAPDH mRNA  in LV tissue | 9.403 | 0.0038 | 0.514 | 0.4774 | 0.183 | 0.6707 |
| Angiotensinogen/GAPDH mRNA in LV tissue | 14.302 | 0.0012 | 0.361 | 0.5549 | 0.176 | 0.6791 |
| Renin/GAPDH mRNA  in LV tissue | 6.430 | 0.0197 | 0.493 | 0.4906 | 0.014 | 0.9077 |
| ACE/GAPDH mRNA  in LV tissue | 13.756 | 0.0014 | 0.339 | 0.5670 | 0.508 | 0.4842 |
| AT_1A_/GAPDH mRNA  in LV tissue | 8.080 | 0.0101 | 1.148 | 0.2967 | 1.779 | 0.1972 |
| Mitochondrial ROS | 8.696 | 0.0122 | 1.586 | 0.2318 | 4.621 | 0.0277 |
| SOD2/GAPDH mRNA  in LV tissue | 56.776 | <0.0001 | 9.676 | 0.0064 | 10.037 | 0.0056 |
| Nox4/GAPDH mRNA  in LV tissue | 7.413 | 0.0151 | 1.669 | 0.2148 | 5.438 | 0.0331 |
| Nox4/GAPDH protein  in LV tissue | 4.855 | 0.0395 | 14.313 | 0.0012 | 5.706 | 0.0269 |
| Mitochondrial area  in LV tissue (μm^2^) | 24.325 | <0.0001 | 2.077 | 0.1650 | 6.521 | 0.0189 |
| Mitochondrial aspect ratio in LV tissue | 26.860 | <0.0001 | 0.098 | 0.7577 | 8.228 | 0.0095 |
| Mitochondrial number  in LV tissue (/µm^2^) | 20.585 | 0.0002 | 0.383 | 0.5431 | 0.008 | 0.9305 |
| Mitochondrial circularity  in LV tissue | 31.158 | <0.0001 | 0.080 | 0.7802 | 10.265 | 0.0045 |
| Mitochondrial form factor  in LV tissue | 26.273 | <0.0001 | 0.166 | 0.6883 | 8.733 | 0.0078 |
| DRP1/GAPDH protein  in LV tissue | 31.296 | <0.0001 | 14.151 | 0.0012 | 25.579 | <0.0001 |
| OPA1/GAPDH protein  in LV tissue | 14.019 | 0.0013 | 3.696 | 0.0689 | 5.199 | 0.0337 |
| NOx in serum (μM) | 19.450 | 0.0003 | 6.877 | 0.0168 | 6.194 | 0.0223 |
| L-Arginine in serum (μM) | 5.070 | 0.0357 | 33.261 | <0.0001 | 3.027 | 0.0973 |
| eNOS/GAPDH mRNA  in LV tissue | 28.859 | <0.0001 | 0.455 | 0.5097 | 0.295 | 0.5944 |
| nNOS/GAPDH mRNA  in LV tissue | 11.474 | 0.0038 | 0.183 | 0.6744 | 2.313 | 0.9881 |
| iNOS/GAPDH mRNA  in LV tissue | 15.441 | 0.0010 | 1.475 | 0.2403 | 0.006 | 0.9378 |
| Arginase I/GAPDH mRNA in LV tissue | 14.812 | 0.0013 | 1.182 | 0.2901 | 0.187 | 0.6706 |
| Arginase II/GAPDH mRNA in LV tissue | 19.798 | 0.0002 | 10.110 | 0.0047 | 8.054 | 0.0102 |
| Arginase activity  in LV tissue (U/L) | 20.563 | 0.0002 | 3.046 | 0.0963 | 5.781 | 0.0260 |
| ODC/GAPDH mRNA  in LV tissue | 8.573 | 0.0127 | 3.456 | 0.0877 | 0.993 | 0.3386 |
| OAT/GAPDH mRNA  in LV tissue | 7.903 | 0.0147 | 5.856 | 0.0309 | 3.895 | 0.0701 |
| GSI | 37.841 | <0.0001 | 0.003 | 0.9579 | 0.168 | 0.6861 |
| CD68-positive cells/glomerulus | 30.647 | <0.0001 | 2.416 | 0.1358 | 0.505 | 0.4856 |
| TIS | 48.363 | <0.0001 | 1.242 | 0.2784 | 0.332 | 0.5708 |
| MCP-1/GAPDH  mRNA in kidney tissue | 21.938 | <0.0001 | 0.048 | 0.8280 | 0.240 | 0.6288 |
| TNF-α/GAPDH  mRNA in kidney tissue | 26.839 | <0.0001 | 0.213 | 0.6484 | 0.047 | 0.8300 |
| COX-2/GAPDH  mRNA in kidney tissue | 17.580 | 0.0003 | 0.160 | 0.6928 | 0.025 | 0.8750 |
| Collagen type I/GAPDH mRNA in kidney tissue | 14.661 | 0.0008 | 0.044 | 0.8349 | 0.759 | 0.3924 |
| Collagen type III/GAPDH mRNA in kidney tissue | 15.208 | 0.0007 | 1.457 | 0.2392 | 0.125 | 0.7270 |
| Collagen type IV/GAPDH mRNA in kidney tissue | 11.038 | 0.0029 | 0.551 | 0.4650 | 0.633 | 0.4341 |
| TGF-β1/GAPDH  mRNA in kidney tissue | 17.374 | 0.0003 | 0.485 | 0.4930 | 0.762 | 0.3912 |
| CTGF/GAPDH  mRNA in kidney tissue | 11.398 | 0.0025 | 0.838 | 0.3692 | 0.771 | 0.3885 |
| Glucose in serum (mg/dL) | 0.023 | 0.8801 | 1.389 | 0.2525 | 0.974 | 0.3356 |
| Total cholesterol in serum (mg/dL) | 10.108 | 0.0047 | 1.075 | 0.3122 | 0.797 | 0.3827 |
| LDL-cholesterol in serum (mg/dL) | 81.256 | <0.0001 | 0.343 | 0.5646 | 0.001 | 0.9768 |
| HDL-cholesterol in serum (mg/dL) | 61.115 | <0.0001 | 0.023 | 0.8803 | 3.781 | 0.9847 |
| Triglyceride in serum (mg/dL) | 26.644 | <0.0001 | 0.418 | 0.5253 | 0.404 | 0.5324 |
| Free fatty acids in serum (mEq/L) | 0.004 | 0.9504 | 0.016 | 0.9006 | 0.147 | 0.7053 |
| Urea nitrogen in serum (mg/dL) | 14.885 | 0.001 | 1.355 | 0.258 | 1.743 | 0.2016 |
| Creatinine in serum (mg/dL) | 10.108 | 0.0047 | 1.075 | 0.3122 | 0.797 | 0.3827 |
| Total protein excretion (mg/day) | 18.275 | 0.0004 | 2.674 | 0.1177 | 0.236 | 0.6324 |
| 24-h CCr (mL min^–1^ g^–1^ kidney) | 64.476 | <0.0001 | 0.851 | 0.3683 | 0.051 | 0.8231 |

**TABLE S2** Serum concentrations of analytes and parameters of renal function for rats of the four experimental groups at 17 weeks of age.

| Parameter | CONT + Ala | CONT + Arg | MetS + Ala | MetS + Arg |
| --- | --- | --- | --- | --- |
| Glucose (mg/dL) | 135.83 ± 7.70 | 142.00 ± 32.58 | 109.00 ± 41.12 | 84.25 ± 25.64 |
| Total cholesterol (mg/dL) | 80.17 ± 15.05 | 85.86 ± 12.47 | 387.20 ± 112.75 | 364.17 ± 160.88 |
| LDL-cholesterol (mg/dL) | 9.00 ± 1.41 | 13.00 ± 5.41 | 59.40 ± 18.96 | 62.50 ± 20.26 |
| HDL-cholesterol (mg/dL) | 28.33 ± 5.35 | 26.50 ± 3.15 | 64.80 ± 16.41 | 64.00 ± 16.01 |
| Triglyceride (mg/dL) | 74.67 ± 19.91 | 76.43 ± 32.39 | 787.20 ± 399.75 | 989.00 ± 675.92 |
| Free fatty acids (mEq/L) | 481.67 ± 55.15 | 511.29 ± 117.99 | 507.60 ± 181.36 | 492.67 ± 183.68 |
| Urea nitrogen (mg/dL) | 24.67 ± 2.37 | 22.89 ± 3.30 | 53.62 ± 21.48 | 81.97 ± 51.89 |
| Creatinine (mg/dL) | 0.44 ± 0.04 | 0.45 ± 0.05 | 0.72 ± 0.35 | 0.95 ± 0.50 |
| Total protein excretion (mg/day) | 77.89 ± 33.79 | 59.93 ± 11.68 | 259.37 ± 95.85 | 264.79 ± 109.76 |
| 24-h CCr (mL min^–1^ g^–1^ kidney) | 0.66 ± 0.14 | 0.73 ± 0.10 | 0.19 ± 0.07 | 0.23 ± 0.22 |

Data are means ± SD, with *n* = 6, 7, 5, and 6 (serum parameters) or *n* = 6, 6, 5, and 5 (urine parameters) for CONT + Ala, CONT + Arg, MetS + Ala, and MetS + Arg groups, respectively.
